# Author Correction: Targeting myeloid derived suppressor cells reverts immune suppression and sensitizes BRAF-mutant papillary thyroid cancer to MAPK inhibitors

**DOI:** 10.1038/s41467-022-34545-6

**Published:** 2022-11-17

**Authors:** Peitao Zhang, Haixia Guan, Shukai Yuan, Huili Cheng, Jian Zheng, Zhenlei Zhang, Yifan Liu, Yang Yu, Zhaowei Meng, Xiangqian Zheng, Li Zhao

**Affiliations:** 1 The Province and Ministry Co-sponsored Collaborative Innovation Center for Medical Epigenetics, Key Laboratory of Immune Microenvironment and Disease (Ministry of Education), Department of Biochemistry and Molecular Biology, School of Basic Medical Sciences, Tianjin Medical University, Tianjin, China; 2 Department of Nuclear Medicine, Tianjin Medical University General Hospital, Tianjin, China; 3 Department of Endocrinology, Guangdong Provincial People’s Hospital, Guangdong Academy of Medical Sciences, Guangzhou, Guangdong Province China; 4 The Second School of Clinical Medicine, Southern Medical University, Guangzhou, Guangdong Province China; 5 Department of Immunology, School of Basic Medical Sciences, Tianjin Medical University, Tianjin, China; 6 Department of Thyroid and Neck Oncology, National Clinical Research Center for Cancer; Key Laboratory of Cancer Prevention and Therapy, Tianjin’s Clinical Research Center for Cancer, Tianjin Medical University Cancer Institute and Hospital, Tianjin, China

**Keywords:** Cancer microenvironment, Thyroid diseases, Tumour immunology

Correction to: *Nature Communications* 10.1038/s41467-022-29000-5, published online 24 March 2022

The original version of this Article contained an error in the author affiliations.

Affiliation 3 incorrectly read ‘Department of Endocrinology, Guangdong Provincial People’s Hospital, Guangdong Academy of Medical Science, Guangzhou, Guangdong Province, China.’

This has now been corrected in both the PDF and HTML versions of the Article.

