# Supplementary material for: Targeting myeloid derived suppressor cells reverts immune suppression and sensitizes BRAF-mutant papillary thyroid cancer to MAPK inhibitors
Source: Nat Commun. 2022 Mar 24;13:1588. doi: 10.1038/s41467-022-29000-5 (PMC8948260; doi:10.1038/s41467-022-29000-5)
Supplement: Supplementary file 1 — Supplementary information [file 41467_2022_29000_MOESM1_ESM.pdf]

Supplementary Information

Targeting myeloid derived suppressor cells reverts immune suppression and sensitizes BRAF-mutant papillary thyroid cancer to MAPK inhibitors

Zhang. et al.

Supplementary Fig. 1

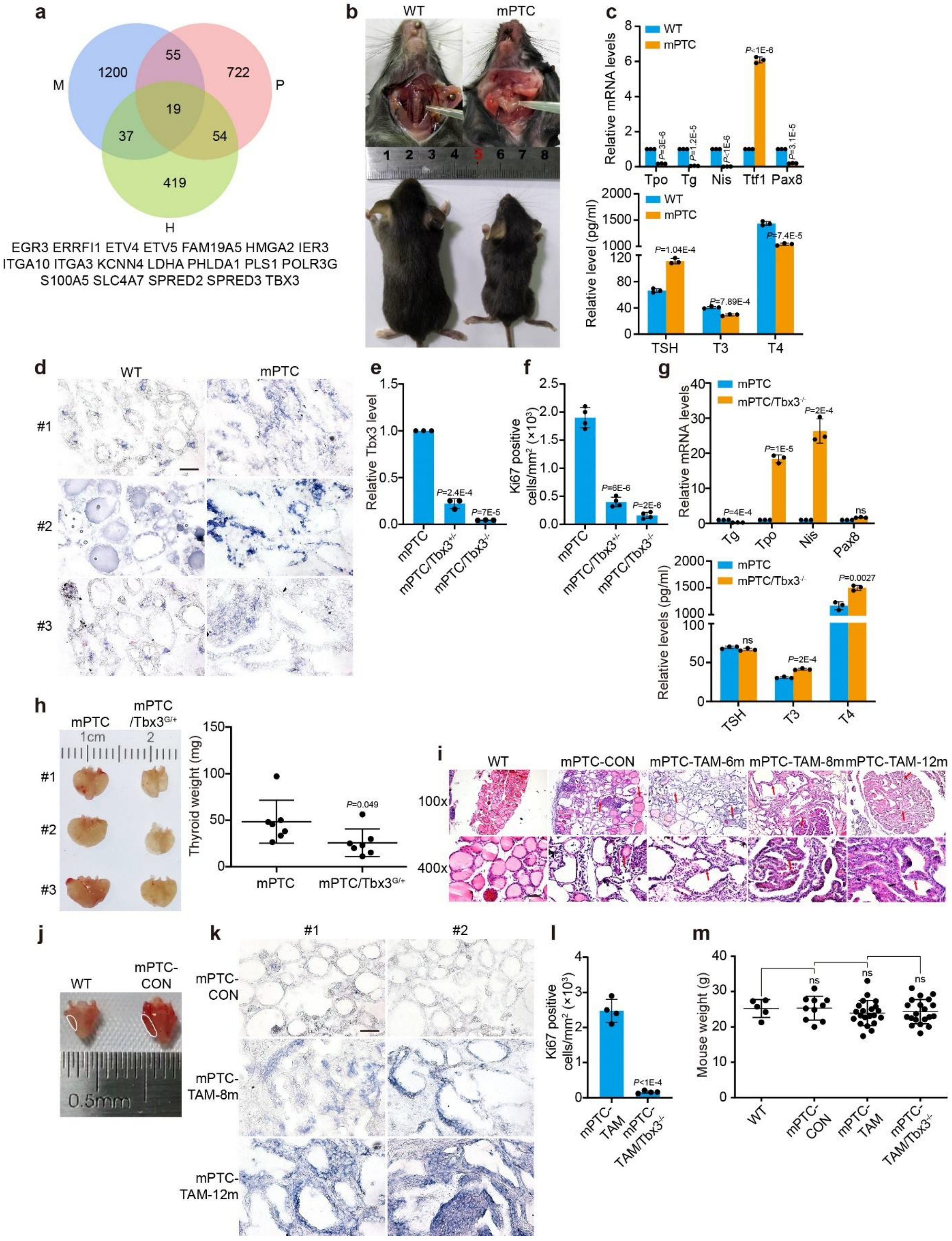

Supplementary Fig. 1 Loss of Tbx3 represses mPTC progression. (**a**) Venn diagram analysis across three groups of genes. M: Down-regulated DEGs in mouse melanoma cells treated with MAPKi (GSE161430); P: Down-regulated DEGs in melanoma patients treated

with MAPKi (GSE75299-patient data part); H: Overlapped down-regulated DEGs from GSE152699 and GSE75299 (cell line data part), referring to human melanoma cell lines treated with MAPKi. (**b**) Gross anatomical images of thyroid tissues from *TPO-cre* (WT) and *TPO-cre*; *LSL-Braf*^{V600E}*CA* (mPTC) at 5w. Corresponding normal and mPTC mice were shown in lower pannel. (**c**) RT-qPCR analysis of thyroid function genes in thyroid tissues from 5w WT and age-matched mPTC littermates. Serum TSH, T3, and T4 levels was measured by ELISA, n=3. (**d**) Representative in-situs of Tbx3 in three pairs of thyroid tissues from WT and mPTC littermates at 5w. Scale bars, 50μm. (**e**) RT-qPCR analysis of Tbx3 in thyroid tissues from WT, mPTC/Tbx3$^{+/-}$, and mPTC/Tbx3$^{-/-}$ mice at 5w. (**f**) The number of positive cells for Ki67 IHC analysis in **Fig. 1e**. (**g**) RT-qPCR analysis of thyroid function genes in thyroid tissues (upper), and ELISA analysis of serum TSH, T3, and T4 levels in 5w mPTC and age-matched mPTC/Tbx3$^{-/-}$ littermates (lower), n=3. (**h**) The images representing whole thyroid tissues from mPTC and mPTC/Tbx3$^{G/+}$ mice at 5w, and the thyroid weight was analyzed, n=7 mPTC, n=7 mPTC/Tbx3$^{G/+}$. (**i**) Representative H&E stainings of *TPO-creER* (WT) and *TPO-creER*; *Braf*^{V600E}*CA* by administration of tamoxifen at 1m (mPTC-TAM) or oil treatment as control (mPTC-CON) for 6m, 8m or 12m. Arrows points to thyroid lobe and papillary architecture. Scale bars, 50 μm. (**j**) Images of thyroid tissues from WT and mPTC-CON referring to **Fig. 1j**, the white line marked the thyroid gland. (**k**) Representative in-situs of Tbx3 in two pairs of thyroid tissues from mPTC-CON, mPTC-TAM-8m, and mPTC-TAM-12m. Scale bars, 50μm. (**l**) The number of positive cells for Ki67 IHC analysis in **Fig. 1i**. (**m**) Mouse weight of WT (n=5), mPTC-CON (n=10), mPTC-TAM (n=21) and mPTC-TAM/Tbx3$^{-/-}$ (n=20) with inducement of tamoxifen for 8m. n=3 biological independent samples (**i**, **k**). Data are shown as the mean ± s.d. (**c**, **e-h**, **l**). P values were calculated by two-tailed Student's t test. ns, no significance. Statistical source data are provided in Source Data.

Supplementary Fig. 2

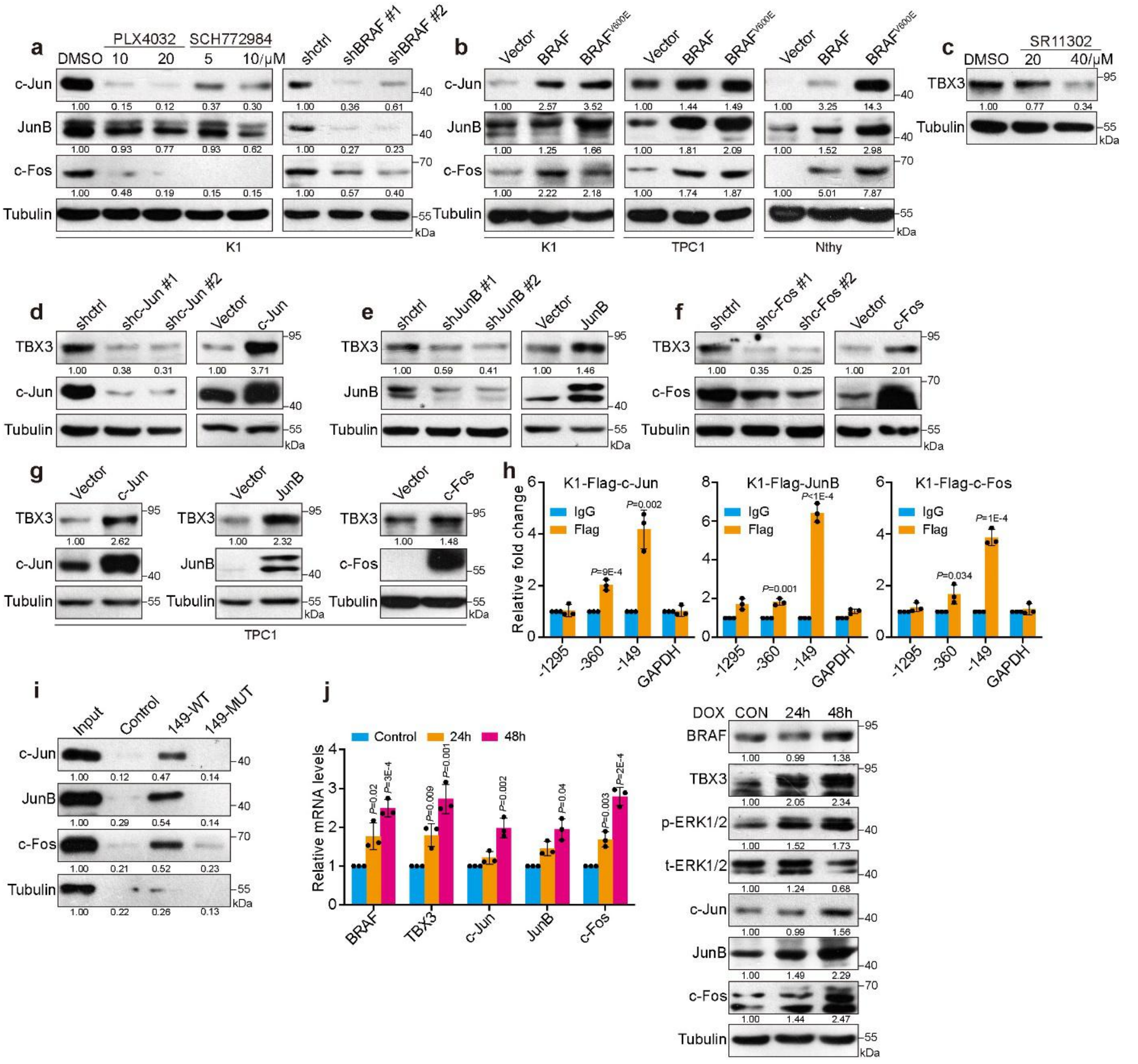

Supplementary Fig. 2 TBX3 is induced by BRAF/MAPK pathway activation. (**a, b**) Western blot of AP-1 factors in PTC or normal thyroid cells treated with PLX4032 or SCH772984 for 24h, or with BRAF knock-down, or with BRAF or BRAFV600E over-expression. (**c**) Western blot of TBX3 in K1 cells treated with AP-1 inhibitor SR11302 for 24h. (**d-f**) Western blot of TBX3 and AP-1 factors in K1 cells with AP-1 knock-down or over-expression. (**g**) Western blot of TBX3 expression in TPC1 cells with AP-1 over-expression. (**h**) Quantitative ChIP (qChIP) analysis with anti-IgG or anti-FLAG for potential AP-1-binding sites in K1 cells stably over-expressed FLAG-tagged AP-1 factors. Results are represented as fold change over control. GAPDH served as a negative control. (**i**) DNA affinity binding

assays were performed using biotin-labeled probe containing −149 site or 149-mut site with K1 nuclear lysates, then analyzed with western blot for AP-1 proteins. (**j**) Expression of TBX3 and AP-1 factors were analysed by RT-qPCR (left) or western blot (right) in TPC1 cells over-expressing $BRAF^{V600E}$ induced by Dox for 24h or 48h. Densitometric analyses of western blot were shown (**a-g**, **i-j**). Two independent experiments were carried out with similar results for each kind of cells (**a-g**), and n=3 biological independent samples (**h-j**). Data are shown as the mean ± s.d. (**h**, **j**). P values were calculated by unpaired two-tailed Student's t test (**h**, **j**). Uncropped immunoblots and statistical source data are provided in Source Data.

Supplementary Fig. 3

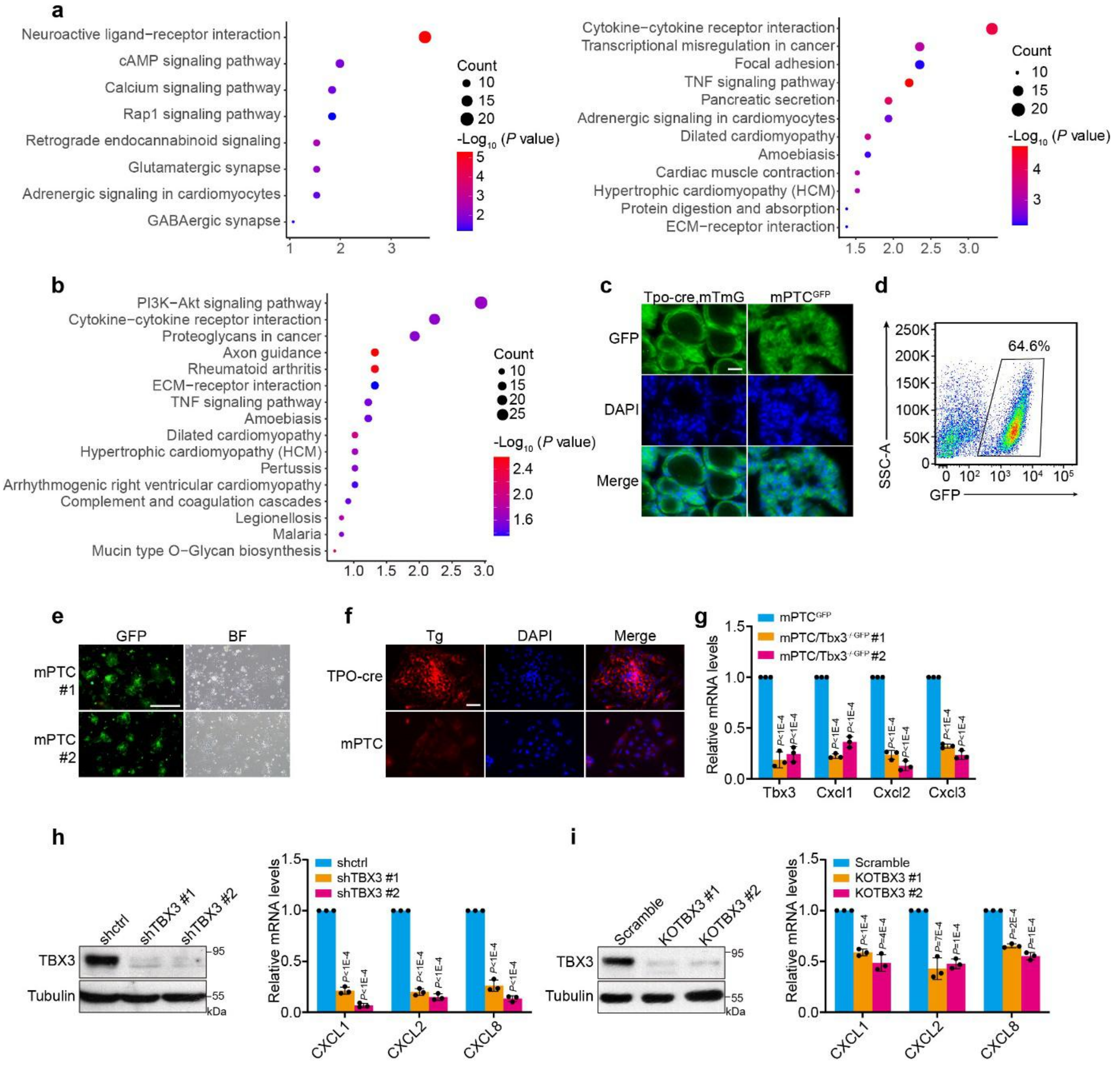

Supplementary Fig. 3 CXCR2 ligands are repressed upon TBX3 loss. (**a**) The KEGG pathway analysis of DEGs according to heatmap (**Fig. 3a**). The left for up-regulated genes, and the right for down-regulated genes, data were analyzed using DAVID 6.8 website. (**b**) The KEGG pathway analysis of down-regulated genes in K1 cells with TBX3 knock-down, data were analyzed using DAVID 6.8 website. **(c)** IF staining of GFP on thyroid gland from *TPO-cre*; *Rosa*26-mTmG and *TPO-cre*; *LSL-Braf^{V600E}CA*; *Rosa26-mTmG* (mPTC^{GFP}) at 5w. Scale bars, 50μm. **(d)** Sorting of GFP^{+} tumor cells from mPTC^{GFP} by flow cytometry. **(e)** Primary culture of mPTC^{GFP} tumor cells 48h post seeding. The pictures were captured in GFP

channel and Bright Field (BF) respectively. Scale bars, 50μm. (**f**) IF staining of Thyroglobulin (Tg) in thyroid gland from *TPO-cre* and mPTC at 5w. Scale bars, 50μm. (**g**) RT-qPCR analysis of Tbx3, Cxcl1, 2, and Cxcl3 in GFP^{+} cells sorting from mPTC and mPTC/$Tbx3^{-/-}$ tumors according to (**d**). (**h**, **i**) RT-qPCR analysis of CXCL1, 2, and CXCL8 in TPC1 cells with TBX3 knock-down (**h**), or K1 cells infected with lentivirus expressing sgRNA targeting TBX3 (**i**). A representative of three independent experiments was shown (**c**, **e-f**), and n=3 biological independent samples (**g-i**). Data are shown as the mean ± s.d. (**g-i**). P values were calculated by unpaired two-tailed Student's t test (**g-i**). Uncropped immunoblots and statistical source data are provided in Source Data.

Supplementary Fig. 4

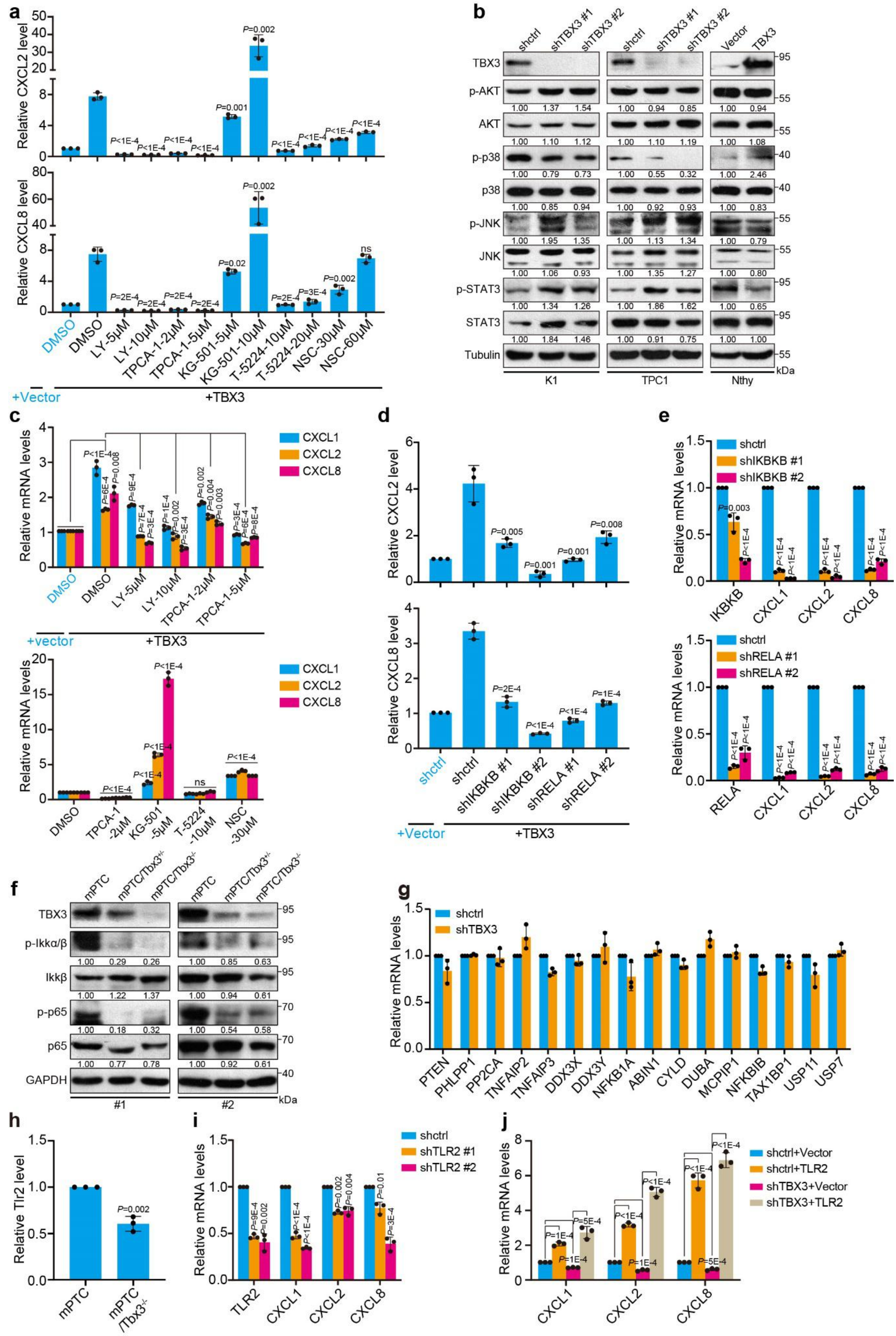

Supplementary Fig. 4 TBX3 promotes chemokines expression in TLR2-NF-κB pathway dependent manner. (**a**) RT-qPCR analysis of CXCL2 and CXCL8 mRNA levels in TBX3 over-expressed Nthy cells treated with small-molecule inhibitors against IKKβ (LY2409881 (LY), TPCA-1), CREB (KG-501), AP-1 (T-5224) or STAT3 (NSC74859 (NSC)). Comparison between DMSO- or inhibitor-treated TBX3-overexpressed Nthy cells were used for statistical analyses respectively, related to **Fig. 4a**. **(b)** Western blot of AKT, p38, JNK, or STAT3 pathway members in K1, TPC1 cells with TBX3 knock-down, and Nthy cells with TBX3 over-expression. (**c**) RT-qPCR analysis of CXCL1, 2 and CXCL8 in TBX3-overexpressed K1 cells treated with LY2409881 or TPCA-1 against IKKβ (upper) and in K1 cells treated with TPCA-1, KG-501, T-5224 or NSC74859 (lower). (**d**) RT-qPCR analysis of CXCL2 and CXCL8 in TBX3 over-expressed Nthy cells infected with shRNAs against IKBKB or RELA, the same comparison was showed as in (**a**), related to **Fig. 4b**. (**e**) RT-qPCR analysis of CXCL1, 2 and CXCL8 in K1 cells infected with shRNAs against IKBKB or RELA. (**f**) Western blot of NF-κB pathway members in thyroid gland lysates from mPTC, mPTC/Tbx3$^{+/-}$ or mPTC/Tbx3$^{-/-}$ littermates. (**g**) RT-qPCR analysis of gene expression in K1 cells infected with shRNA against TBX3. (**h**) RT-qPCR analysis of Tlr2 in tumors from mPTC/Tbx3$^{-/-}$ compared with mPTC. (**i**) mRNA expression of TLR2, CXCL1, 2 and CXCL8 in K1 cells infected with shRNAs against TLR2. (**j**) mRNA expression of CXCL1, 2 and CXCL8 in K1 cells with TBX3 knock-down and/or TLR2 over-expression. Densitometric analyses of western blot were shown (**b**, **f**). *n*=3 biological independent samples (**a**, **c-j**), and two independent experiments were carried out with similar results for each kind of cells (**b**). Data are shown as the mean ± s.d. (**a**, **c-e**, **g-j**). *P* values were calculated by unpaired two-tailed Student's *t* test (**a**, **c-e**, **g-j**). Uncropped immunoblots and statistical source data are provided in Source Data.

Supplementary Fig. 5

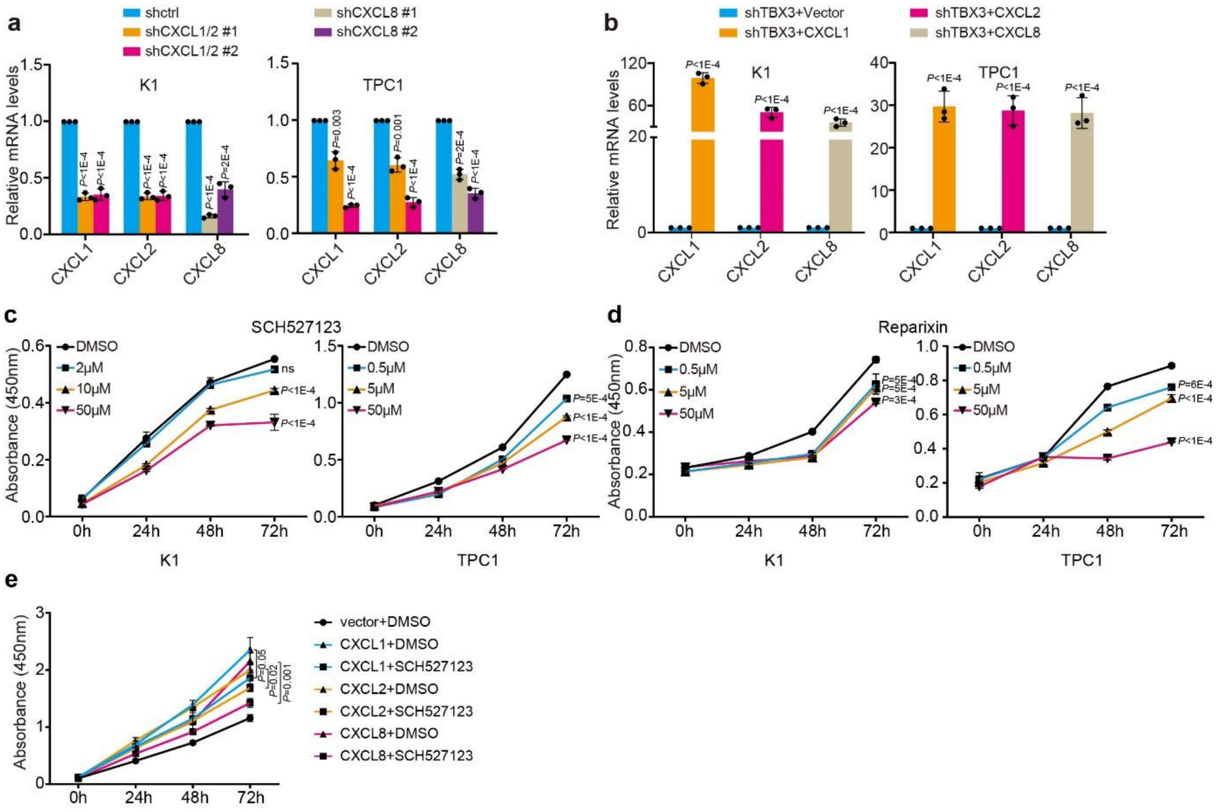

Supplementary Fig. 5 CXCR2 ligands promote PTC cell proliferation in an autocrine manner. (**a**) mRNA levels of CXCL1, 2 and CXCL8 were checked by RT-qPCR in PTC cells infected with lentivirus expressing specific shRNA against CXCL1/2 or CXCL8. (**b**) mRNA levels of CXCL1, 2 and CXCL8 were checked by RT-qPCR in TBX3 knocked-down PTC cells with or without CXCLs over-expression. (**c, d**) Effect of CXCR2 blocked by CXCR2 inhibitor SCH527123 (**c**) or Reparixin (**d**) on PTC cells proliferation was analyzed. (**e**) CCK8 assays were performed in K1 cells over-expressing CXCL1, 2 and CXCL8, and treated with DMSO or SCH527123 at a concentration of 50uM. *n*=3 biological independent samples (**a-e**). Data are shown as the mean ± s.d. (**a-e**). *P* values were calculated by unpaired two-tailed Student's *t* test (**a-e**). Statistical source data are provided in Source Data.

Supplementary Fig. 6

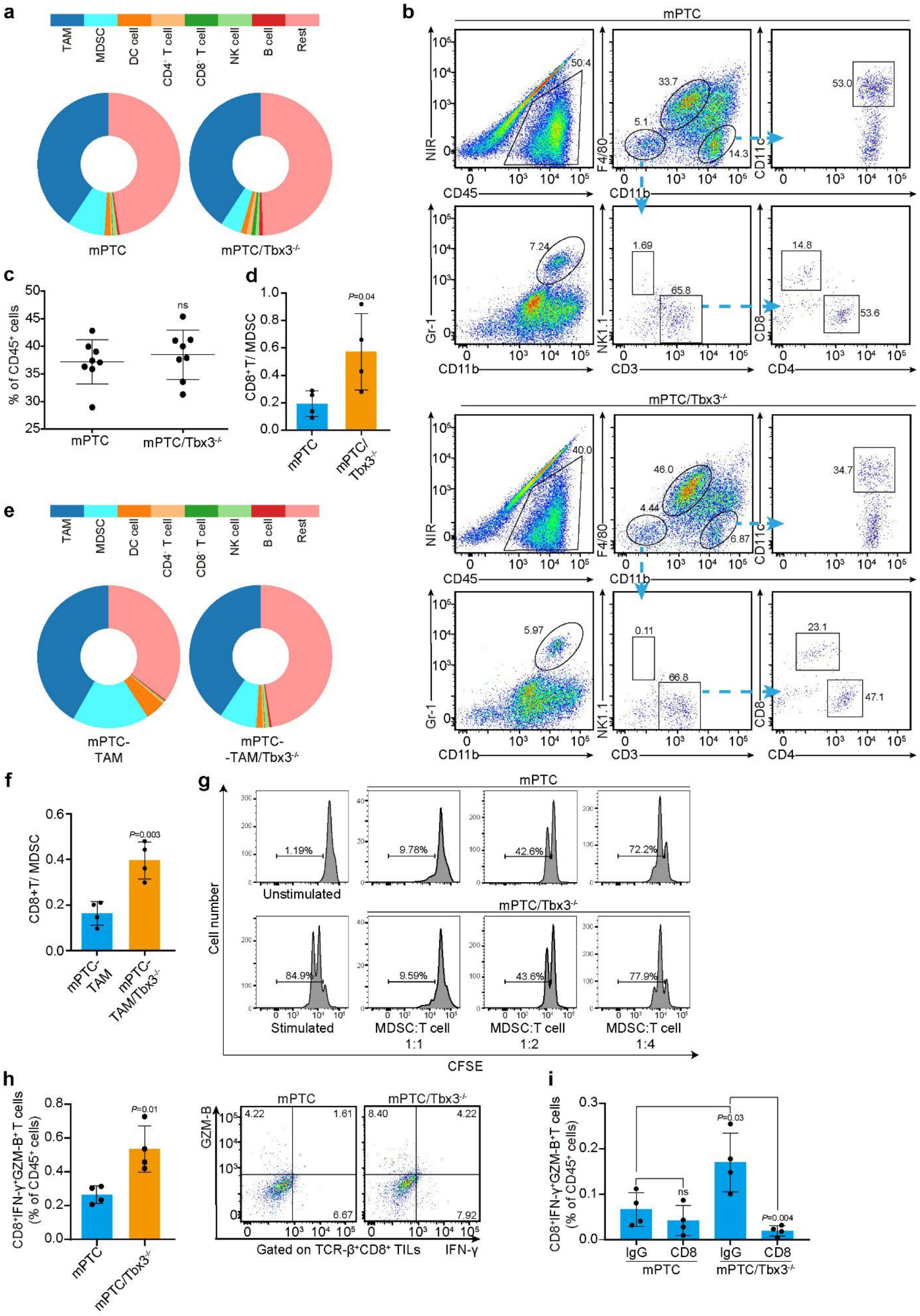

Supplementary Fig. 6 Loss of Tbx3 in thyroid cancer represses immune-suppression. (**a**, **b**) Quantification of whole leucocyte populations from mPTC and mPTC/Tbx3$^{-/-}$ littermates by FlowJo (**a**). Immune cells were identified as tumor-associated macrophages (TAM) (CD45^{+}CD11b^{+}F4/80^{+}), MDSCs (CD45^{+}CD11b^{+}Gr-1^{+}), dendritic cells (DC) (CD45^{+}F4/80^{-}CD11b^{+}CD11c^{+}), CD4^{+} T cells (CD45^{+}CD3^{+}NK1.1^{-}CD4^{+}CD8^{-}), CD8^{+} T cells (CD45^{+}CD3^{+}NK1.1^{-}CD4^{-}CD8^{+}), B cells (CD45^{+}CD3^{-}CD19^{+}), natural killer (NK) cells (CD45^{+}CD3^{-}NK1.1^{+}) (**b**), *n*=4. (**c**) Percentage of TAM of CD45^{+} TILs in mPTC tumors compared with mPTC/Tbx3$^{-/-}$ littermates at 5w was analyzed by flow cytometry, *n*=8. (**d**) Relative CD8^{+} T cell: total MDSC ratio was plotted, referring to **Fig. 6c**, *n*=4. (**e**) Flow cytometric analysis of whole leucocyte populations from mPTC-TAM compared with mPTC-TAM/Tbx3$^{-/-}$ littermates induced with tamoxifen for 8m, *n*=4. (**f**) Relative CD8^{+} T cells: total MDSCs ratio was plotted, referring to **Fig. 6f**, *n*=4. (**g**) Flow cytometric analysis of the CFSE-labeled T cell proliferation activated with T-activator CD3/28, co-cultured with MDSCs isolated from mPTC and mPTC/Tbx3$^{-/-}$ tumors. The percentage of proliferating CD8^{+} T cells is indicated in each panel. A representative of three independent experiments was shown. (**h**) Percentage of IFN-γ- and Granzyme B (GZM-B)-expressing, activated CD8^{+} T cells of total CD45^{+} TILs from mPTC and mPTC/Tbx3$^{-/-}$ at 5w was analyzed by FlowJo, *n*=4. (**i**) Percentage of CD8^{+}IFN-γ^{+}GZM-B^{+} T cells of total CD45^{+} TILs in tumors was analyzed by FlowJo, referring to **Fig. 6g**, *n*=4. Data are shown as the mean ± s.d. (**c-d**, **f**, **h-i**). *P* values were calculated by unpaired two-tailed Student's *t* test (**d**, **f**, **h-i**). Statistical source data are provided in Source Data.

Supplementary Fig. 7

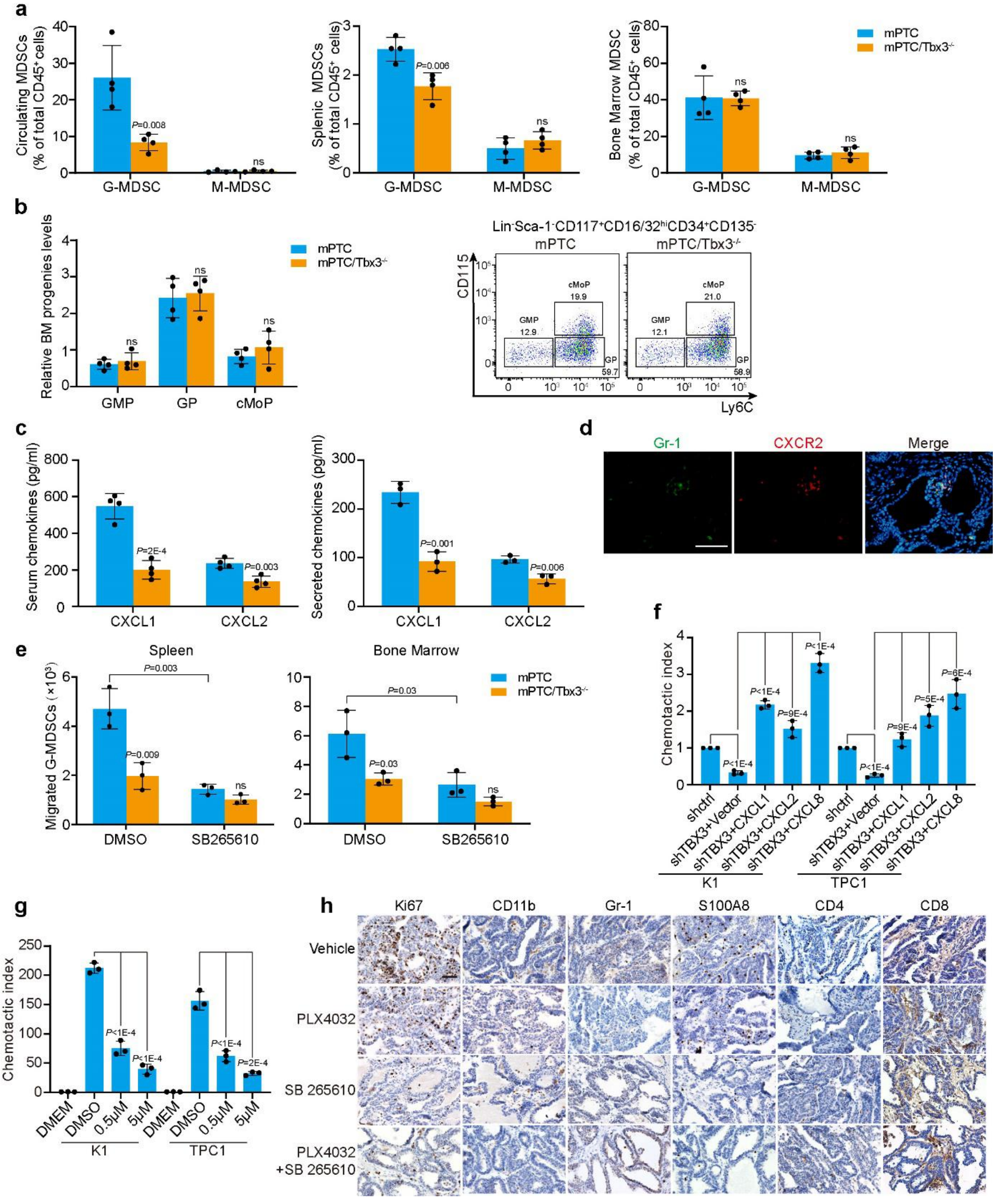

Supplementary Fig. 7 Tbx3/Cxcr2 ligands axis promotes MDSCs recruitment in thyroid cancer. (**a**) Percentage of G-MDSCs and M-MDSCs of total CD45$^+$ leukocytes in the peripheral blood, spleen and bone marrow (BM) from mPTC and mPTC/Tbx3$^{-/-}$ mice was analyzed by FlowJo, *n*=4. (**b**) Flow cytometric analysis of GMPs (Granulocyte-monocyte

progenitors, Lin$^-$Sca-1$^-$CD117$^+$CD16/32^{hi}CD34$^+$CD135$^-$Ly6C$^-$CD115$^-$), GPs (Granulocyte progenitors, Lin$^-$Sca-1$^-$CD117$^+$CD16/32^{hi}CD34$^+$CD135$^-$Ly6C$^+$CD115$^-$) and cMoPs (Common monocyte progenitor, Lin$^-$Sca-1$^-$CD117$^+$CD16/32^{hi}CD34$^+$CD135$^-$Ly6C$^+$CD115$^+$) of BM progenitors from mPTC and mPTC/Tbx3$^{-/-}$ mice at age of 5w according to the sorting strategy of Zhaoyuan Liu (Cell, 2019)[1], *n*=4. (**c**) Levels of CXCL1 and CXCL2 in blood (left, *n*=4) and supernatants of primary tumor cells (right, *n*=4) were measured by ELISA. (**d**) Expression and localization of Gr-1 and CXCR2 in PTC tissues were analyzed by IF staining. Scale bars, 100μm. (**e**) Migration of spleen and BM G-MDSCs toward conditioned medium from cultured primary mPTC and mPTC/Tbx3$^{-/-}$ cells, which were treated with SB265610 at 5μM, was evaluated through 3mm-transwell migration assay. *n*=3. (**f**) Neutrophil chemotaxis through 3mm-transwell migration assay was detected in response to the conditioned media from TBX3 knock-down PTC cells with Vector and CXCLs over-expression. (**g**) Neutrophil chemotaxis was detected in response to the conditioned media from PTC cells treated SCH527123 at 0.5 and 5μM. (**h**) Representative images of IHC staining in four groups of mPTC were shown, referring to **Fig. 6h**. Scale bars, 50μm. A representative of three independent experiments was shown (**d**). *n*=3 biological independent samples (**f**, **g**). Data are shown as the mean ± s.d (**a**, **c**, **e-g**). *P* values were calculated by unpaired two-tailed Student's *t* test (**a**, **c**, **e-g**). Statistical source data are provided in Source Data.

Supplementary Fig. 8

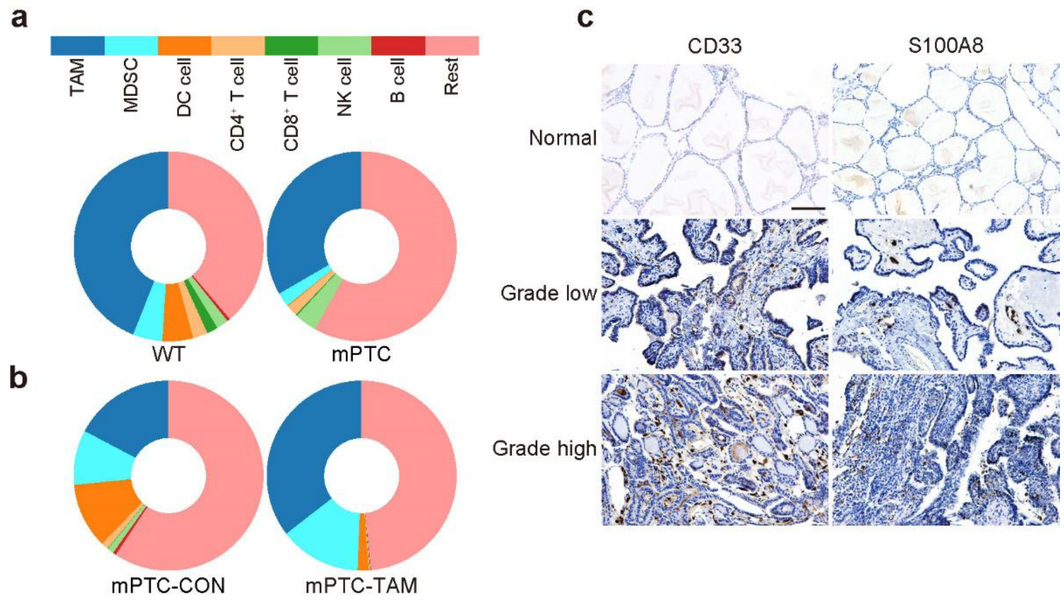

Supplementary Fig. 8 The infiltration of MDSCs increases in PTC. (**a**) Quantification of whole leucocyte populations in thyroid glands from mPTC and control littermates by FlowJo, *n*=5. (**b**) Quantification of whole leucocyte populations in thyroid glands from mPTC-CON and mPTC-TAM induced with oil or tamoxifen for 8m by FlowJo, *n*=4. (**c**) IHC staining of CD33 and S100A8 on PTC samples (histological Grades low [I, II] and Grades high [III and IV]) paired with adjacent normal tissues. A representative of three independent experiments was shown. Scale bars, 100μm. Statistical source data are provided in Source Data.

Supplementary Fig. 9

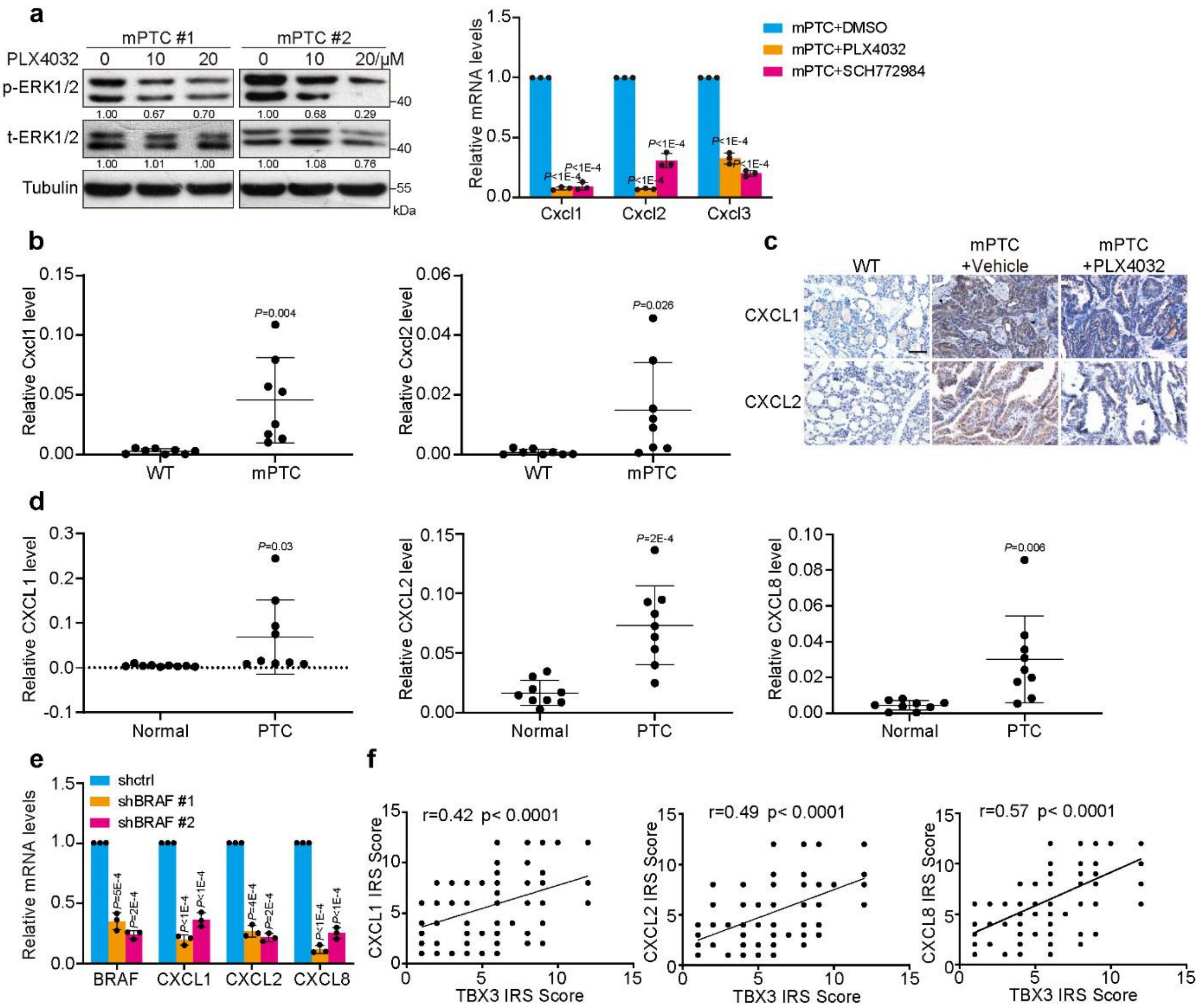

Supplementary Fig. 9 CXCR2 ligands are highly expressed in BRAF^{V600E} mutation PTC. (**a**) Expression of p-ERK1/2 and ERK1/2 in mPTC^{GFP} tumor cells treated with PLX4032 was checked by western blot (left), and the levels of Cxcl1, Cxcl2 and Cxcl3 in mPTC^{GFP} tumor cells treated with PLX4032 or SCH772984 were checked by RT-qPCR (right). (**b**) RT-qPCR analysis of mRNA levels of Cxcl1 and Cxcl2 in thyroid glands from mPTC (*n*=8) and age-matched wild-type littermates (*n*=8) at 5w. (**c**) IHC analysis of CXCL1 and CXCL2 in WT and mPTC treated with PLX4032 in **Fig. 6h**. Scale bars, 50μm. (**d**) RT-qPCR analysis mRNA levels of CXCL1, 2 and CXCL8 in human PTC tissues (*n*=9) and paracancerous tissues (Normal, *n*=9). (**e**) Levels of CXCLs in K1 cells with BRAF knockdown were checked by RT-qPCR. (**f**) IHC results from **Fig. 8e** were used to analyze the correlation coefficient between

TBX3 and CXCL1, CXCL2 and CXCL8. *n*=3 biological independent samples (**a**, **c**, **e**). Data are shown as the mean ± s.d (**a**-**b**, **d**-**f**). *P* values were calculated by unpaired two-tailed Student's *t* test (**a**-**b**, **d**-**f**). Uncropped immunoblots and statistical source data are provided in Source Data.

Supplementary Table 1. List of shRNA sequences

shctrl	CCTAAGGTTAAGTCGCCCTCG
shTBX3 #1	GCGAATGTTTCCTCCATTTAA
shTBX3 #2	GCTGATGACTGTCGTTATAAA
shBRAF #1	TCTGGAGGCCTATGAAGAATA
shBRAF #2	GACCTCAAGAGTAATAATATA
shc-Jun #1	AGATGGAAACGACCTTCTATG
shc-Jun #2	GAAAGTCATGAACCACGTTAA
shJunB #1	ACGACTACAAACTCCTGAAAC
shJunB #2	TCATACACAGCTACGGGATAC
shc-Fos #1	TCTGCTTTGCAGACCGAGATT
shc-Fos #2	GCGGAGACAGACCAACTAGAA
shCXCL1/2 #1	AGATGCTGAACAGTGACAAAT
shCXCL1/2 #2	CGAAGTCATAGCCACACTCAA
shCXCL8 #1	ACTTAGATGTCAGTGCATAAA
shCXCL8 #2	TATCCAGAACATACTTATATG
shIKBKB #1	CCAGCCAAGAAGAGTGAAGAA
shIKBKB #2	GCTGGTTCATATCTTGAACAT
shRELA #1	CGGATTGAGGAGAAACGTAAA
shRELA #2	CCTGAGGCTATAACTCGCCTA
shTLR2 #1	TTTGATGACTGTACCCTTAAT
shTLR2 #2	ACTTATCCAGCACACGAATAC

Supplementary Table 2. List of qPCR primers

Human gene	Strand	Sequence
TBX3	F	TTTGAAGACCATGGAGCCCG
TBX3	R	ACATTCGCCTTCCCGACTTG
BRAF	F	AGACGGGACTCGAGTGATGA
BRAF	R	AACTGCTGAGGTGTAGGTGC
c-Jun	F	GTGCCGAAAAAGGAAGCTGG
c-Jun	R	CTGCGTTAGCATGAGTTGGC
JunB	F	GTCAAAGCCCTGGACGATCT
JunB	R	TTGGTGTAAACGGGAGGTGG
c-Fos	F	GGGGCAAGGTGGAACAGTTA
c-Fos	R	AGGTTGGCAATCTCGGTCTG
FosB	F	GCTGCAAGATCCCCTACGAAG
FosB	R	ACGAAGAAGTGTACGAAGGGTT
CXCL1	F	GCGCCCAAACCGAAGTC
CXCL1	R	TGCAGGATTGAGGCAAGCTT
CXCL2	F	CTGCGCCCAAACCGAAGTCATA
CXCL2	R	TCAGTTGGATTTGCCATTTT
CXCL3	F	CCCAAACCGAAGTCATAGCC
CXCL3	R	AGTTCCCCACCCTGTCATTT
CXCL8	F	CCTGATTTCTGCAGCTCTGT
CXCL8	R	AACTTCTCCACAACCCTCTG
IL-10	F	TCAAGGCGCATGTGAACTCC
IL-10	R	GATGTCAAACTCACTCATGGCT
Oncostatin	F	CACAGACTGGCCGACTTAGAG
Oncostatin	R	AGTCCTCGATGTTCAGCCCA
GCSF	F	GCTGCTTGAGCCAACTCCATA
GCSF	R	GAACGCGGTACGACACCTC
GM-CSF	F	TCCTGAACCTGAGTAGAGACAC
GM-CSF	R	TGCTGCTTGTAGTGGCTGG
CCL-7	F	ATCCCTAAGCAGAGGCTGGA
CCL-7	R	GTCCTGGACCCACTTCTGTG
TGFB1	F	GGCCAGATCCTGTCCAAGC
TGFB1	R	GTGGGTTTCCACCATTAGCAC
PDGF-BB	F	CTCGATCCGCTCCTTTGATGA
PDGF-BB	R	CGTTGGTGCGGTCTATGAG
PTEN	F	GCTATGGGATTTCCTGCAG
PTEN	R	CTAGCTGTGGTGGGTTATGG
PHLPP1	F	GTCAAAGCCCTGGACGATCT
PHLPP1	R	TTGGTGTAAACGGGAGGTGG
PP2CA	F	GCCTACAAGAAGTTCCCCATGAG
PP2CA	R	TCTGCGAGAAGGCTAAAGA
TNFAIP2	F	GCTGACGCGGCTTTCCCG
TNFAIP2	R	GCTCCTCCTCGCTCACACCG
TNFAIP3	F	TCCTCAGGCTTTGTATTTGAGC
TNFAIP3	R	TGTGTATCGGTGCATGGTTTTA
DDX3X	F	CCGCAAACAATACCCAATCT
DDX3X	R	CCACGCAAGGACGAACTCTA

DDX3Y	F	GGAACAGAGAAGCATCTAAAGG
DDX3Y	R	CATCATAGTCACTCCGTCCA
NFKB1A	F	GCCGCTCCTTCTTCAGCC
NFKB1A	R	AGCCCACAGCAGTCCGTG
ABIN1	F	CTAGTGTGACGGCAGGTAAGG
ABIN1	R	GCTGCTTCATGGACCGGAA
CYLD	F	TTCACTGACGGGGTGTACCA
CYLD	R	CAGGACCTGCGTAATCACTTTC
DUBA	F	GGTTGTGCGAAAGCATTGCAT
DUBA	R	ACCTCCACAGGACGGTTGT
MCPIP1	F	ACGGGATCGTGGTTTCCAAC
MCPIP1	R	TGGCTTCTTACGCAGGAAGTT
NFKBIB	F	GCTGACCTTGACAAACCGGA
NFKBIB	R	GCCGGATTTCTCGTCCTCG
TAX1BP1	F	AAGAAACAGCACAACTTCGAGA
TAX1BP1	R	TGGATGTAGCATCACTGAACCT
USP11	F	TATAAGCAGTGGGAGGCATACG
USP11	R	ATGACCTTGCGTTCAATGGGT
USP7	F	GGAAGCGGGAGATACAGATGA
USP7	R	AAGGACCGACTCACTCAGTCT
IKBKB	F	GGAAGTACCTGAACCAGTTTGAG
IKBKB	R	GCAGGACGATGTTTTCTGGCT
RELA	F	CTATAGAAGAGCAGCGTGGGG
RELA	R	TCACTCGGCAGATCTTGAGC
TLR2	F	TTATCCAGCACACGAATACACAG
TLR2	R	AGGCATCTGGTAGAGTCATCAA
Ki67	F	TGAGCCTGTACGGCTAAAACA
Ki67	R	GGCCTTGGAATCTTGAGCTTT
GAPDH	F	CCTGTTCGACAGTCAGCCG
GAPDH	R	CGACCAAATCCGTTGACTCC
**Mouse gene**	**Strand**	**sequence**
Tbx3	F	CTGGCAATGGCAGGAGAGAA
Tbx3	R	GAGACAGCAGGAGAGGATGC
Tlr2	F	GCAAACGCTGTTCTGCTCAG
Tlr2	R	AGGCGTCTCCCTCTATTGTATT
Cxcl1	F	CCTATCGCCAATGAGCTGCG
Cxcl1	R	CCTCGCGACCATTCTTGAGT
Cxcl2	F	GAAGACCCTGCCAAGGGTTG
Cxcl2	R	AGGCAAACTTTTTGACCGCC
Cxcl3	F	GAACACCCTACCAAGGGTTGA
Cxcl3	R	TCAGCTGGACTTGCCGC
Tnfα	F	GTAGCCCACGTCGTAGCAAA
Tnfα	R	TAGCAAATCGGCTGACGGTG
Tnfβ	F	CACACGAGGTCCAGCTCTTT
Tnfβ	R	GTAGATGGGAGATGCCGTCG
Il-1α	F	GAGCCGGGTGACAGTATCAG
Il-1α	R	ACTTCTGCCTGACGAGCTTC
Il1r2	F	GTTTCTGCTTTCACCACTCCA
Il1r2	R	GAGTCCAATTTACTCCAGGTCAG

Ccl-7	F	ATAGCCGCTGCTTTCAGCAT
Ccl-7	R	CTCGACCCACTTCTGATGGG
Tgfβ-1	F	AGCTGCGCTTGCAGAGATTA
Tgfβ-1	R	AGCCCTGTATTCCGTCTCCT
Gm-csf	F	CTCACCCATCACTGTCACCC
Gm-csf	R	AAATTGCCCCGTAGACCCTG
S100a8	F	AAATCACCATGCCCTCTACAAG
S100a8	R	CCCACTTTTATCACCATCGCAA
S100a9	F	ATACTCTAGGAAGGAAGGACACC
S100a9	R	TCCATGATGTCATTTATGAGGGC
Tpo	F	TGACTTCCAGGAGCACACAG
Tpo	R	GCAAGTTCAGTGATGCCAGA
Tg	F	TGTCCCACCAAGTGTGAAAA
Tg	R	CCAAGGAAAGCTTGTTCAGC
Nis	F	GCTCAGTCTCGCTCAAAACC
Nis	R	CGTGTGACAGGCCACATAAC
Ttf1	F	TCCAGCCTATCCCATCTGAACT
Ttf1	R	CAAGCGCATCTCACGTCTCA
Pax8	F	CAGAAGGCGTTTGTGACAATGA
Pax8	R	TGCACTTTGGTCCGGATGAT
Igfbp3	F	CACACCGAGTGACCGATTCC
Igfbp3	R	GTGTCTGTGCTTTGAGACTCAT
Cdkn1c	F	CGAGGAGCAGGACGAGAATC
Cdkn1c	R	GAAGAAGTCGTTCGCATTGGC
Cdkn2c	F	GGGGACCTAGAGCAACTTACT
Cdkn2c	R	AAATTGGGATTAGCACCTCTGAG
Mmp9	F	CTGGACAGCCAGACACTAAAG
Mmp9	R	CTCGCGGCAAGTCTTCAGAG
Klf9	F	GCCGCCTACATGGACTTCG
Klf9	R	GGTCACCGTGTTCCTTGGT
Met	F	GTGAACATGAAGTATCAGCTCCC
Met	R	TGTAGTTTGTGGCTCCGAGAT
Six4	F	CCACGGTTTTTCCCTGACCC
Six4	R	GGTTGCATAGTTAGTGTTGCTGA
Gapdh	F	AGGTCGGTGTGAACGGATTTG
Gapdh	R	TGTAGACCATGTAGTTGAGGTCA
**ChIP-qPCR**	**Strand**	**sequence**
GAPDH	F	GAAGGCTGGGGCTCATTT
GAPDH	R	CAGGAGGCATTGCTGATGAT
-149	F	GTTACAGTTTCCGACACCTT
-149	R	CCTCTTGATTGGCTCTTTGA
-360	F	GGAGTGCAAGAGAGGCGAGC
-360	R	CTCGCTCGCTCTGGTTCAGC
-1295	F	TGGGTGCACAGCACAGGGTAA
-1295	R	AAAGCGAGCACAGCGAGAGA

References

1 Liu, Z. et al. Fate Mapping via Ms4a3-Expression History Traces Monocyte-Derived Cells. *Cell* **178**, 1509-1525 e1519 (2019).
