# Supplementary material for: Targeting myeloid derived suppressor cells reverts immune suppression and sensitizes BRAF-mutant papillary thyroid cancer to MAPK inhibitors
Source: Nat Commun. 2022 Mar 24;13:1588. doi: 10.1038/s41467-022-29000-5 (PMC8948260; doi:10.1038/s41467-022-29000-5)
Supplement: Supplementary file 2 — Reporting Summary [file 41467_2022_29000_MOESM2_ESM.pdf]

nature research

Corresponding author(s): Li Zhao

Last updated by author(s): Feb 10, 2022

Reporting Summary

Nature Research wishes to improve the reproducibility of the work that we publish. This form provides structure for consistency and transparency in reporting. For further information on Nature Research policies, see our Editorial Policies and the Editorial Policy Checklist.

Statistics

For all statistical analyses, confirm that the following items are present in the figure legend, table legend, main text, or Methods section.

n/a	Confirmed	
☐	☒	The exact sample size (*n*) for each experimental group/condition, given as a discrete number and unit of measurement
☐	☒	A statement on whether measurements were taken from distinct samples or whether the same sample was measured repeatedly
☐	☒	The statistical test(s) used AND whether they are one- or two-sided<br>*Only common tests should be described solely by name; describe more complex techniques in the Methods section.*
☒	☐	A description of all covariates tested
☒	☐	A description of any assumptions or corrections, such as tests of normality and adjustment for multiple comparisons
☐	☒	A full description of the statistical parameters including central tendency (e.g. means) or other basic estimates (e.g. regression coefficient) AND variation (e.g. standard deviation) or associated estimates of uncertainty (e.g. confidence intervals)
☐	☒	For null hypothesis testing, the test statistic (e.g. *F*, *t*, *r*) with confidence intervals, effect sizes, degrees of freedom and *P* value noted<br>*Give P values as exact values whenever suitable.*
☒	☐	For Bayesian analysis, information on the choice of priors and Markov chain Monte Carlo settings
☒	☐	For hierarchical and complex designs, identification of the appropriate level for tests and full reporting of outcomes
☐	☒	Estimates of effect sizes (e.g. Cohen's *d*, Pearson's *r*), indicating how they were calculated

Our web collection on statistics for biologists contains articles on many of the points above.

Software and code

Policy information about availability of computer code

Data collection	qRT-PCR data was collected by LightCycler 480 real-time PCR system (Roche); Flow cytometry was performed on LSRFortessa analyzer (Becton Dickinson); Fluorescent images were acquired using LeicaDMI3000B fluorescence microscopy.
Data analysis	Cluster 3.0 and DAVID 6.8 (https://david.ncifcrf.gov/tools.jsp) were used for analyses of RNA-seq data; FlowJo V10 was used for flow cytometry data ; SPSS 17.0 and GraphPad Prism 8.0 were used for statistical analyses.

For manuscripts utilizing custom algorithms or software that are central to the research but not yet described in published literature, software must be made available to editors and reviewers. We strongly encourage code deposition in a community repository (e.g. GitHub). See the Nature Research guidelines for submitting code & software for further information.

Data

Policy information about availability of data

All manuscripts must include a data availability statement. This statement should provide the following information, where applicable:
- Accession codes, unique identifiers, or web links for publicly available datasets
- A list of figures that have associated raw data
- A description of any restrictions on data availability

Jaspar database(http://jaspar.genereg.net).
GEO datasets (GEO Accession Number: GSE126153, GSE126154, GSE161430, GSE75299 and GSE152699) and TCGA-THCA dataset available from tcga.org are used for analyses in this study.
The RNA-seq data generated in this study have been deposited in the Gene Expression Omnibus (GEO) under the accession GSE166513.
All data associated with this study are present in the paper or the Supplementary information. Source data are provided with this paper.

Field-specific reporting

Please select the one below that is the best fit for your research. If you are not sure, read the appropriate sections before making your selection.

- [x] Life sciences
- [ ] Behavioural & social sciences
- [ ] Ecological, evolutionary & environmental sciences

For a reference copy of the document with all sections, see nature.com/documents/nr-reporting-summary-flat.pdf

Life sciences study design

All studies must disclose on these points even when the disclosure is negative.

Sample size	The sample size for each experiment is specified in the Methods section and figure legends. The sample size was determined by relevant published research or previous experience with similar type of experiments (Saqcena, M. et al. Cancer Discov (2020). Li, X. M. et al. Oncogene 37, 2773-2792 (2018).) to confirm a result with statistically significant.
Data exclusions	For tumor formation experiments and inhibitor treatment in mouse assays (Fig. 5e, Fig.6g-h and Fig. 7i), the highest and lowest tumor of each group were rexcluded from analysis according to the pre-established exclusion criteria.
Replication	For each experiment, the number of biological replicates is reported in the figure legend.
Randomization	All cell samples were randomly allocated to experimental and control groups. In each mice experimental series, animals were assigned to various groups in random according to planned age and weight.
Blinding	In each mice experimental series, the administration of treatments and the subsequent analysis were performed by different operator. The immunohistochemical analysis of the PTC was finished by two pathologists who did not know each other. For the other cell experiments, two investigators performed with different cell lines, respectively. And data analysis was blinded to the experimental conditions.

Reporting for specific materials, systems and methods

We require information from authors about some types of materials, experimental systems and methods used in many studies. Here, indicate whether each material, system or method listed is relevant to your study. If you are not sure if a list item applies to your research, read the appropriate section before selecting a response.

Materials & experimental systems

n/a	Involved in the study	
[ ]	[x]	Antibodies
[ ]	[x]	Eukaryotic cell lines
[x]	[ ]	Palaeontology and archaeology
[ ]	[x]	Animals and other organisms
[ ]	[x]	Human research participants
[x]	[ ]	Clinical data
[x]	[ ]	Dual use research of concern

Methods

n/a	Involved in the study	
[x]	[ ]	ChIP-seq
[ ]	[x]	Flow cytometry
[x]	[ ]	MRI-based neuroimaging

Antibodies

Antibodies used

All antibodies used in the study are reported in Methods.

TBX3 Polyclonal Antibody Invitrogen Cat# 42-4800
Monoclonal Anti-a-Tubulin Simga Cat# T5168 Clone B-5-1-2
B-Raf (D9T6S) Rabbit mAb CST Cat# 14814 Clone D9T6S
c-Jun (60A8) Rabbit mAb CST Cat# 9165 Clone 60A8
p44/42 MAPK (Erk1/2) (137F5) Rabbit mAb CST Cat# 4695 Clone 137F5
Phospho-p44/42 MAPK (Erk1/2) (Thr202/Tyr204) (D13.14.4E) XP® Rabbit mAb CST Cat# 4370 Clone D13.14.4E
FosB (5G4) Rabbit mAb CST Cat# 2251 Clone 5G4
JunB (C37F9) Rabbit mAb CST Cat# 3753 Clone C37F9
c-Fos (9F6) Rabbit mAb CST Cat# 2250 Clone 9F6
GAPDH (D16H11) XP® Rabbit mAb CST Cat# 5174 Clone D16H11
β-Actin (8H10D10) Mouse mAb CST Cat# 3700 Clone 8H10D10
NF-κB p65 (D14E12) XP® Rabbit mAb CST Cat# 8242 Clone D14E12
Phospho-NF-κB p65 (Ser536) (93H1) Rabbit mAb CST Cat# 3033 Clone 93H1
IKKβ (D30C6) Rabbit mAb CST Cat# 8943 Clone D30C6
Phospho-IKKα/β (Ser176/180) (16A6) Rabbit mAb CST Cat# 2697 Clone 16A6
TLR2 Polyclonal Antibody Bioss Cat# bs-1019R

Akt (pan) (C67E7) Rabbit mAb CST Cat# 4691 Clone C67E7
Phospho-Akt (Ser473) (D9E) XP® Rabbit mAb CST Cat# 4060 Clone D9E
p38 MAPK (D13E1) XP® Rabbit mAb CST Cat# 8690 Clone D13E1
Phospho-p38 MAPK (Thr180/Tyr182) (D3F9) XP® Rabbit mAb CST Cat# 4511 Clone D3F9
SAPK/JNK Antibody CST Cat# 9252
Phospho-SAPK/JNK (Thr183/Tyr185) (G9) Mouse mAb CST Cat# 9255 Clone G9
Stat3 (124H6) Mouse mAb CST Cat# 9139 Clone 124H6
Phospho-Stat3 (Tyr705) (D3A7) XP® Rabbit mAb CST Cat# 9145 Clone D3A7
Anti-Ki67 antibody Abcam Cat# ab16667 Clone SP6
CXCL1/GRO alpha antibody GeneTex Cat# GTX31184
CXCL2/GRO beta antibody GeneTex Cat# GTX31171
Anti-Thyroglobulin antibody Abcam Cat# ab156008 Clone EPR9730
TBX3 Polyclonal Antibody Proteintech Cat# 16741-1-AP
CD8α (D4W2Z) XP® Rabbit mAb (Mouse Specific) CST Cat# 98941 Clone D4W2Z
Anti-CD4 antibody Abcam Cat# ab183685 Clone EPR19514
Anti- MRP8 antibody Abcam Cat# ab92331 Clone EPR3554
Purified anti-mouse Ly-6G/Ly-6C (Gr-1) Antibody Biolegend Cat# 108401 Clone RB6-8C5
Anti-CD11b antibody Abcam Cat# ab133357 Clone EPR1344
IL-8 Polyclonal Antibody ImmunoWay Cat# T5153
Alexa Fluor® 700 anti-mouse CD45 Antibody Biolegend Cat# 147716 Clone I3/2.3
FITC anti-mouse CD45 Antibody Biolegend Cat# 103108 Clone 30-F11
PE anti-mouse F4/80 Antibody Biolegend Cat# 123110 Clone BM8
Pacific Blue™ anti-mouse/human CD11b Antibody Biolegend Cat# 101224 Clone M1/70
FITC anti-mouse CD3ε Antibody Biolegend Cat# 100306 Clone 145-2C11
PerCP/Cyanine5.5 anti-mouse CD3ε Antibody Biolegend Cat# 100328 Clone 145-2C11
APC anti-mouse NK-1.1 Antibody Biolegend Cat# 108709 Clone PK136
Brilliant Violet 605™ anti-mouse CD4 Antibody Biolegend Cat# 100451 Clone GK1.5
Alexa Fluor® 700 anti-mouse CD4 Antibody Biolegend Cat# 100430 Clone GK1.5
Brilliant Violet 510™ anti-mouse CD8a Antibody Biolegend Cat# 100752 Clone 53-6.7
PerCP/Cyanine5.5 anti-mouse Ly-6G/Ly-6C (Gr-1) Antibody Biolegend Cat# 108428 Clone RB6-8C5
Brilliant Violet 605™ anti-mouse Ly-6G Antibody Biolegend Cat# 127639 Clone 1A8
Brilliant Violet 510™ anti-mouse Ly-6C Antibody Biolegend Cat# 128033 Clone HK1.4
PE/Dazzle™ 594 anti-mouse CD11c Antibody Biolegend Cat# 117347 Clone N418
PE/Cyanine7 anti-mouse CD19 Antibody Biolegend Cat# 115519 Clone 6D5
Purified Rat Anti-Mouse CD16/CD32 (Mouse BD Fc Block™) Becton Dickinson Cat# 553141 Clone 2.4G2
PE-Cy7 anti-Ly-6C Antibody Biolegend Cat# 128017 Clone HK1.4
APC anti-CD115 Antibody Biolegend Cat# 135509 Clone AFS98
PE anti-CD34 Antibody Biolegend Cat# 152203 Clone SA376A4
PE-CF594 anti-CD135 Antibody Biolegend Cat# 313319 Clone BV10A4H2
BV421 anti-CD117 Antibody Biolegend Cat# 105827 Clone 2B8
FITC anti-CD16/32 Antibody Biolegend Cat# 101305 Clone 93
AF-700 anti-Sca-1 Antibody Biolegend Cat# 108141 Clone D7
Percp-cy5.5 anti-Ter-119 Antibody Biolegend Cat# 116227 Clone TER-119
APC anti-IFN-γ Antibody Biolegend Cat# 505809 Clone XMG1.2
PE anti- Granzyme B Antibody Biolegend Cat# 96405 Clone QA18A28
Anti-Digoxigenin-AP antibody Roche Cat# 11093274910
InVivoMAb rat IgG2a isotype control, anti-trinitrophenol Bio-XCell Cat# BE0089 Clone 2A3
InVivoMAb anti-mouse Ly6G/Ly6C (Gr-1) Bio-XCell Cat# BE0075 Clone RB6-8C5
InVivoMAb anti-mouse CD8 Bio-XCell Cat# BE0061 Clone 2.43

Validation

All antibodies were obtained from commercial sources with reported validation, and we confirmed with negative and positive control before the study.
Abbreviation for species cross reactivity: H-human, M-mouse, R-rat, Hm-hamster, Mk-monkey, Vir-virus, Mi-mink, C-chicken, Dm-Drosophila melanogaster, X-xenopus, Z-zebrafish, B-bovine, Dg-dog, PG-pig, Sc-Saccharomyces cerevisiae, Ce-Caenorhabditis elegans, Hr-horse, K-Kangaroo, Su-Sea urchin, Ch-Chlamydomonas, S-Sheep

TBX3 Polyclonal Antibody Invitrogen Cat# 42-4800 H, M, R PMID: 27110270
Monoclonal Anti-a-Tubulin Simga Cat# T5168 Clone B-5-1-2 C, K, R, SU, R, CH, B, H, MK, M PMID: 31523176
B-Raf (D9T6S) Rabbit mAb CST Cat# 14814 Clone D9T6S H, M, R, M PMID: 34992144
c-Jun (60A8) Rabbit mAb CST Cat# 9165 Clone 60A8 H, M, R, M PMID: 35027468
p44/42 MAPK (Erk1/2) (137F5) Rabbit mAb CST Cat# 4695 Clone 137F5 H, M, R, Hm, Mk, Mi, Dm, Z, B, Dg, Pg, Ce PMID: 35028009
Phospho-p44/42 MAPK (Erk1/2) (Thr202/Tyr204) (D13.14.4E) XP® Rabbit mAb CST Cat# 4370 Clone D13.14.4E H, M, R, Hm, Mk, Mi, Dm, Z, B, Dg, Pg, Sc PMID: 34965411
FosB (5G4) Rabbit mAb CST Cat# 2251 Clone 5G4 H, M, R PMID: 34352786
JunB (C37F9) Rabbit mAb CST Cat# 3753 Clone C37F9 H, M, R, M PMID: 34744629
c-Fos (9F6) Rabbit mAb CST Cat# 2250 Clone 9F6 H, M, R PMID: 34673574
GAPDH (D16H11) XP® Rabbit mAb CST Cat# 5174 Clone D16H11 H, M, R, Mk PMID: 34955649
β-Actin (8H10D10) Mouse mAb CST Cat# 3700 Clone 8H10D10 H, M, R, Hm, Mk, Dg PMID: 34977268
NF-κB p65 (D14E12) XP® Rabbit mAb CST Cat# 8242 Clone D14E12 H, M, R, Hm, Mk, Dg PMID: 34558536

Phospho-NF-κB p65 (Ser536) (93H1) Rabbit mAb CST Cat# 3033 Clone 93H1 H, M, R, Hm, Mk, Pg PMID: 34901529
IKKβ (D30C6) Rabbit mAb CST Cat# 8943 Clone D30C6 H, M, R, Hm, Mk PMID: 33971703
Phospho-IKKα/β (Ser176/180) (16A6) Rabbit mAb CST Cat# 2697 Clone 16A6 H, M, R, Hm, Mk PMID: 35002723
TLR2 Polyclonal Antibody Bioss Cat# bs-1019R H, M, R, B, Pg, S PMID: 23295061
Akt (pan) (C67E7) Rabbit mAb CST Cat# 4691 Clone C67E7 H, M, R, Mk, Dm, PMID: 35005567
Phospho-Akt (Ser473) (D9E) XP® Rabbit mAb CST Cat# 4060 Clone D9E H, M, R, Hm, Mk, Dm, Z, B PMID: 35024764
p38 MAPK (D13E1) XP® Rabbit mAb CST Cat# 8690 Clone D13E1 H, M, R, Hm, Mk, B, Pg PMID: 34935053
Phospho-p38 MAPK (Thr180/Tyr182) (D3F9) XP® Rabbit mAb CST Cat# 4511 Clone D3F9 H, M, R, Mk, Mi, Pg, Sc PMID: 34935053
SAPK/JNK Antibody CST Cat# 9252 H, M, R, Hm, Mk, Z, B, Sc PMID: 35027468
Phospho-SAPK/JNK (Thr183/Tyr185) (G9) Mouse mAb CST Cat# 9255 Clone G9 H, M, R, Hm, Sc PMID: 35028009
Stat3 (124H6) Mouse mAb CST Cat# 9139 Clone 124H6 H, M, R, Mk PMID: 35020440
Phospho-Stat3 (Tyr705) (D3A7) XP® Rabbit mAb CST Cat# 9145 Clone D3A7 H, M, R, Mk PMID: 34938610
Anti-Ki67 antibody Abcam Cat# ab16667 Clone SP6 H, M, R PMID: 34284046
CXCL1/GRO alpha antibody GeneTex Cat# GTX31184 validated in the manufacturers' websites.
CXCL2/GRO beta antibody GeneTex Cat# GTX31171 validated in the manufacturers' websites.
Anti-Thyroglobulin antibody Abcam Cat# ab156008 Clone EPR9730 H, M, R PMID: 33162555
TBX3 Polyclonal Antibody Proteintech Cat# 16741-1-AP H, M PMID: 29620145
CD8α (D4W2Z) XP® Rabbit mAb (Mouse Specific) CST Cat# 98941 Clone D4W2Z M PMID: 34952899
Anti-CD4 antibody Abcam Cat# ab183685 Clone EPR19514 M PMID: 32401602
Anti- MRP8 antibody Abcam Cat# ab92331 Clone EPR3554 H, M PMID: 33301706
Purified anti-mouse Ly-6G/Ly-6C (Gr-1) Antibody Biolegend Cat# 108401 Clone RB6-8C5 M PMID: 22965162
Anti-CD11b antibody Abcam Cat# ab133357 Clone EPR1344 H, M, R PMID: 33283987
IL-8 Polyclonal Antibody ImmunoWay Cat# T5153 H PMID: 28033248
Anti-Digoxigenin-AP antibody Roche Cat# 11093274910 PMID: 24318810
InVivoMAb rat IgG2a isotype control, anti-trinitrophenol Bio-XCell Cat# BE0089 Clone 2A3 PMID: 30097293
InVivoMAb anti-mouse Ly6G/Ly6C (Gr-1) Bio-XCell Cat# BE0075 Clone RB6-8C5 PMID: 29311363
InVivoMAb anti-mouse CD8 Bio-XCell Cat# BE0061 Clone 2.43 PMID: 27775706

The following antibodies for Flow Cytometry were all validated in the manufacturers' websites with a large number of research.
Alexa Fluor® 700 anti-mouse CD45 Antibody Biolegend Cat# 147716 Clone I3/2.3
FITC anti-mouse CD45 Antibody Biolegend Cat# 103108 Clone 30-F11
PE anti-mouse F4/80 Antibody Biolegend Cat# 123110 Clone BM8
Pacific Blue™ anti-mouse/human CD11b Antibody Biolegend Cat# 101224 Clone M1/70
FITC anti-mouse CD3ε Antibody Biolegend Cat# 100306 Clone 145-2C11
PerCP/Cyanine5.5 anti-mouse CD3ε Antibody Biolegend Cat# 100328 Clone 145-2C11
APC anti-mouse NK-1.1 Antibody Biolegend Cat# 108709 Clone PK136
Brilliant Violet 605™ anti-mouse CD4 Antibody Biolegend Cat# 100451 Clone GK1.5
Alexa Fluor® 700 anti-mouse CD4 Antibody Biolegend Cat# 100430 Clone GK1.5
Brilliant Violet 510™ anti-mouse CD8a Antibody Biolegend Cat# 100752 Clone 53-6.7
PerCP/Cyanine5.5 anti-mouse Ly-6G/Ly-6C (Gr-1) Antibody Biolegend Cat# 108428 Clone RB6-8C5
Brilliant Violet 605™ anti-mouse Ly-6G Antibody Biolegend Cat# 127639 Clone 1A8
Brilliant Violet 510™ anti-mouse Ly-6C Antibody Biolegend Cat# 128033 Clone HK1.4
PE/Dazzle™ 594 anti-mouse CD11c Antibody Biolegend Cat# 117347 Clone N418
PE/Cyanine7 anti-mouse CD19 Antibody Biolegend Cat# 115519 Clone 6D5
Purified Rat Anti-Mouse CD16/CD32 (Mouse BD Fc Block™) Becton Dickinson Cat# 553141 Clone 2.4G2
PE-Cy7 anti-Ly-6C Antibody Biolegend Cat# 128017 Clone HK1.4
APC anti-CD115 Antibody Biolegend Cat# 135509 Clone AFS98
PE anti-CD34 Antibody Biolegend Cat# 152203 Clone SA376A4
PE-CF594 anti-CD135 Antibody Biolegend Cat# 313319 Clone BV10A4H2
BV421 anti-CD117 Antibody Biolegend Cat# 105827 Clone 2B8
FITC anti-CD16/32 Antibody Biolegend Cat# 101305 Clone 93
AF-700 anti-Sca-1 Antibody Biolegend Cat# 108141 Clone D7
Percp-cy5.5 anti-Ter-119 Antibody Biolegend Cat# 116227 Clone TER-119
APC anti-IFN-γ Antibody Biolegend Cat# 505809 Clone XMG1.2
PE anti- Granzyme B Antibody Biolegend Cat# 396405 Clone QA18A28.

Eukaryotic cell lines

Policy information about cell lines

Cell line source(s)	HEK293T (ACS-4500) was purchased from the American Type Culture Collection (ATCC). And all cancer cell lines were obtained from Tianjin Medical University Cancer Institute and Hospital with STR profiling.
Authentication	Cell lines have been confirmed with STR Authentication.
Mycoplasma contamination	All cell lines have tested for mycoplasma and found to be negative
Commonly misidentified lines (See ICLAC register)	We did not use commonly misidentified lines.

Animals and other organisms

Policy information about studies involving animals; ARRIVE guidelines recommended for reporting animal research

Laboratory animals	And BALB/c nude mouse of female at 5-6w at the beginning of study. C57BL/6 strain including Tpo-cre, LSL-BrafV600ECA, Tbx3flox/flox mice , Tbx3-GFP, TPO-creER , Rosa-mTmG of both sexes at 4w-12m. All mice were maintained under SPF-condition at normal room temperature with a 12/12 h light/dark cycle and normal ambient humidity.
Wild animals	No wild animals were used.
Field-collected samples	The study did not involve field-collected samples.
Ethics oversight	All mouse experiment procedures and protocols were evaluated and authorized by the Regulations of Tianjin Laboratory Animal Management and strictly followed the guidelines under the Institutional Animal Care and Use Committee of Tianjin Medical University .

Note that full information on the approval of the study protocol must also be provided in the manuscript.

Human research participants

Policy information about studies involving human research participants

Population characteristics	PTC samples were collected from (1) 10-30 years old including 3 men and 19 women; (2) 30-50 years old including 16 men and 59 women; and (3) >50 years old including 5 men and 38 women. Fresh samples from adjacent normal tissues, pathological grade I, II, III and IV were frozen in liquid nitrogen immediately after resection or fixed in 4% paraformaldehyde (Sigma Aldrich) at 4℃ overnight before embedded in paraffin. Patients with a past history of radiation therapy or chemotherapy were excluded from this study. The healthy volunteers used in neutrophil chemotaxis assays were 25-30 years old including 2 men and 2 women.
Recruitment	Recruitment participants were recruited by the doctors participating in the study at Tianjin cancer institute and hospital (Tianjin, China).Tumor tissue samples were collected according to standard clinical procedures. The study did not look at the results of individual patients, nor did the doctors or patients involved in the study rule out bias. In addition, the healthy volunteers in this study were recruited with normal certificate of physical examination and no selection bias was observed.
Ethics oversight	All PTC samples were obtained from Tianjin cancer institute and hospital, and the patients signed an informed consent form issued by the 'Ethics Committee of Tianjin Cancer Institute and Hospital'. In addition, healthy volunteers signed informed consent issued by the 'Ethics Committee of Tianjin Cancer Institute and Hospital' before recruitment for the study.

Note that full information on the approval of the study protocol must also be provided in the manuscript.

Flow Cytometry

Plots

Confirm that:

- [x] The axis labels state the marker and fluorochrome used (e.g. CD4-FITC).
- [x] The axis scales are clearly visible. Include numbers along axes only for bottom left plot of group (a 'group' is an analysis of identical markers).
- [x] All plots are contour plots with outliers or pseudocolor plots.
- [x] A numerical value for number of cells or percentage (with statistics) is provided.

Methodology

Sample preparation	Mouse PTC tumors were minced into small fragments on ice and then separated into single cells with tissue dissociation buffer including 1mg/ml collagenase I (Sigma) and 0.5 mg/ml dispase II (Invitrogen) dissolved in PBS at 37℃ for 60-90min. Samples were mashed through 40μm filters into FACS buffer (1% FBS in PBS) and washed for three times.The single cells were stained with Zombie NIR™ Fixable Viability Kit (#423105, Biolegend) for 15min in dark on ice, and then incubated with anti-CD16/CD32 (#553141, Becton Dickinson) for blocking and other indicated antibodies for 30 min on ice.
Instrument	LSRFortessa analyzer (Becton Dickinson).
Software	FlowJo V10 Software.
Cell population abundance	The purity of the population after sort was more than 95%.
Gating strategy	First eliminate duplex, gate on size and granularity to narrow down specific populations using FSC/SSC; and then live/dead discrimination with NIR signal; finally, set gates with indicated staining.

- [x] Tick this box to confirm that a figure exemplifying the gating strategy is provided in the Supplementary Information.
