# Supplementary material for: Targeting myeloid derived suppressor cells reverts immune suppression and sensitizes BRAF-mutant papillary thyroid cancer to MAPK inhibitors
Source: Nat Commun. 2022 Mar 24;13:1588. doi: 10.1038/s41467-022-29000-5 (PMC8948260; doi:10.1038/s41467-022-29000-5)
Supplement: Supplementary file 7 — Description of Additional Supplementary Files [file 41467_2022_29000_MOESM7_ESM.pdf]

Title: Supplementary Data 1.

Description: List of the overlapped DEGs from the RNA-sequencing analysis data of human and mouse.

Title: Supplementary Data 2.

Description: List of the chemokines from analysis of antibody array on K1 and TPC1 cells with TBX3 knock-down.

Title: Supplementary Data 3.

Description: List of the overlapped DEGs from the RNA-sequencing analysis data of PTC and breast cancer cells.
