# Supplementary material for: Targeting myeloid derived suppressor cells reverts immune suppression and sensitizes BRAF-mutant papillary thyroid cancer to MAPK inhibitors
Source: Nat Commun. 2022 Mar 24;13:1588. doi: 10.1038/s41467-022-29000-5 (PMC8948260; doi:10.1038/s41467-022-29000-5)

Fig. 1c

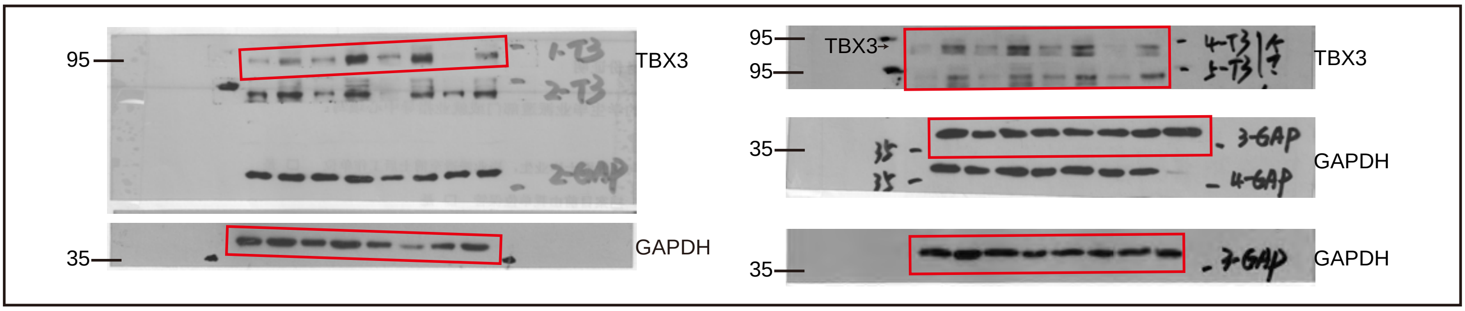

Fig. 2a

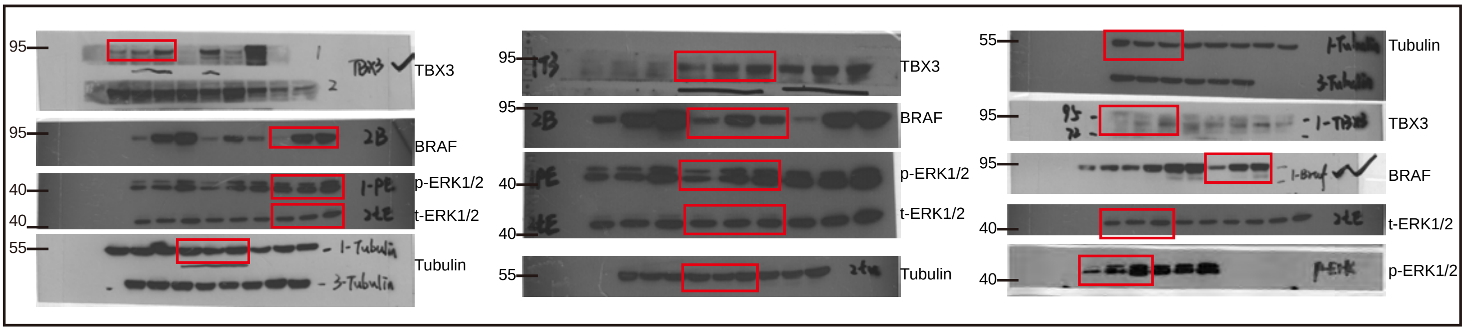

Fig. 2b

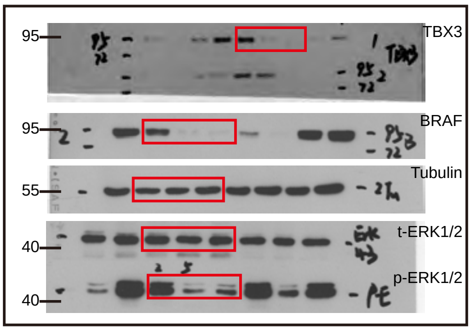

Fig. 2c

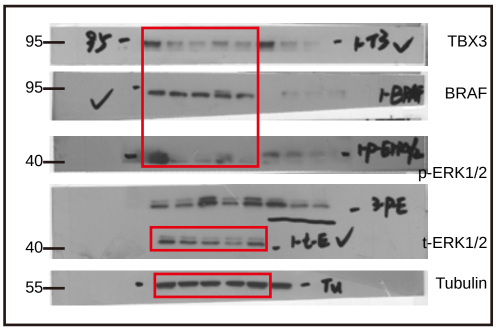

Fig. 2d

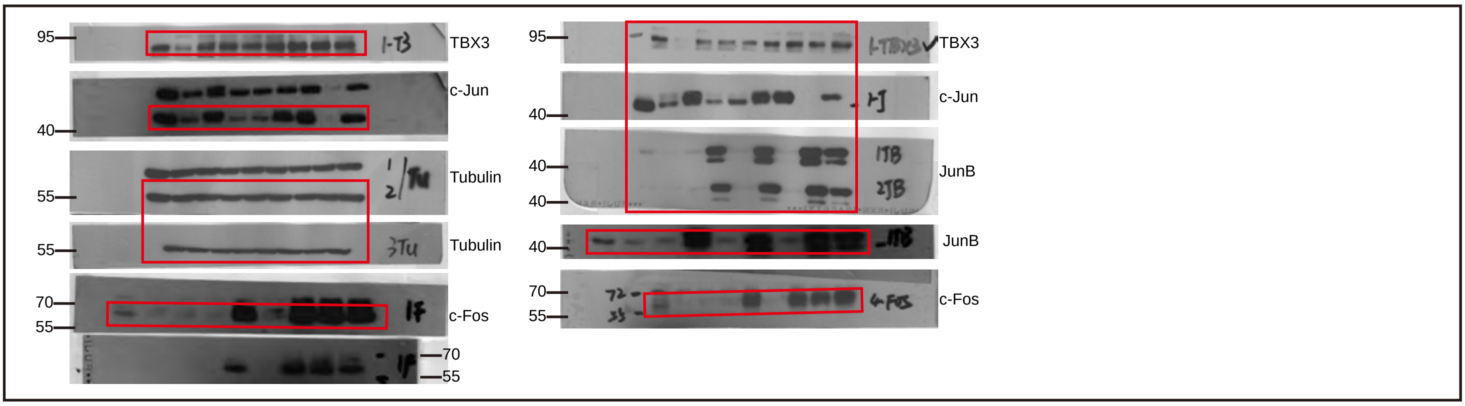

Fig. 2e

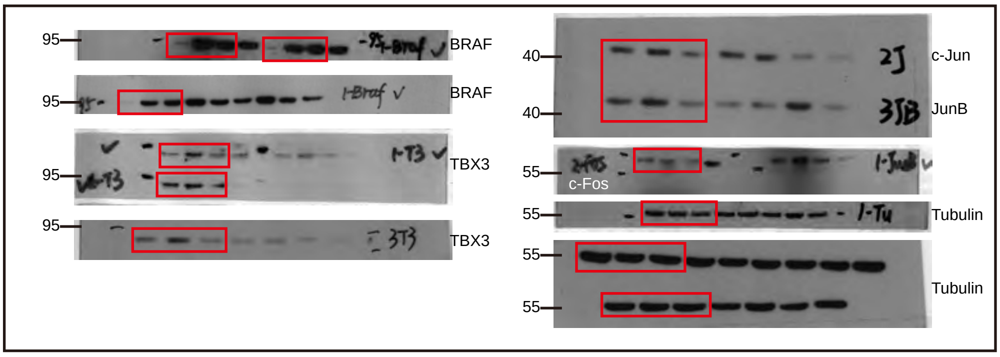

Fig. 3h

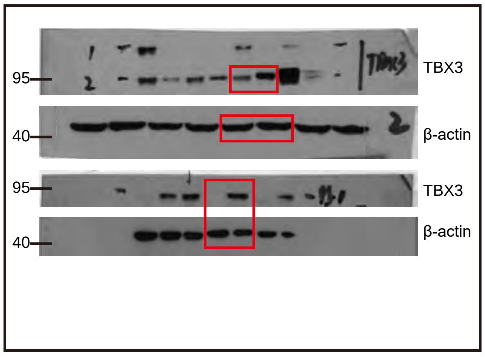

Fig. 4c

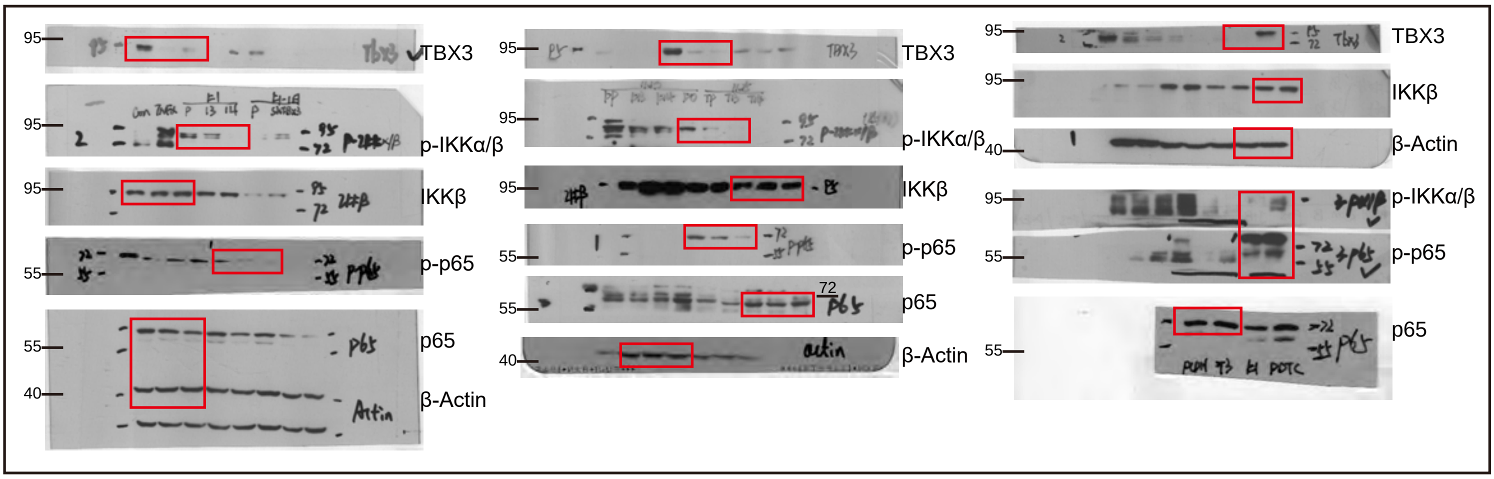

Fig. 4g

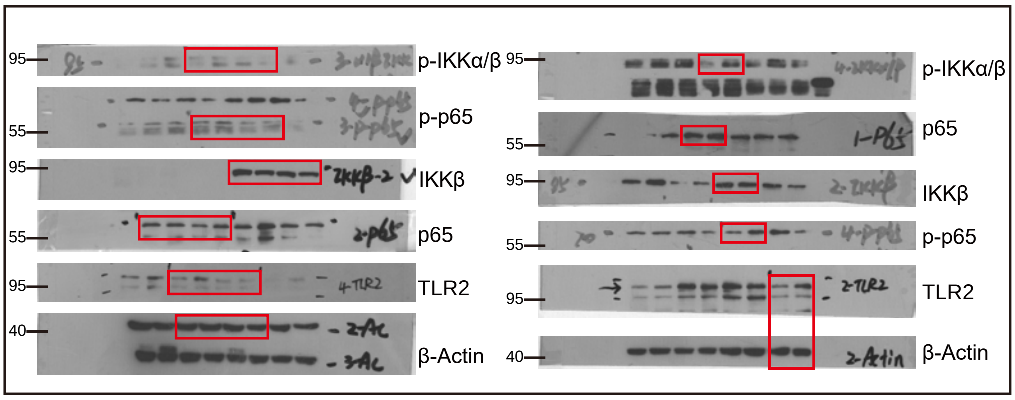

Supplementary Fig. 2a

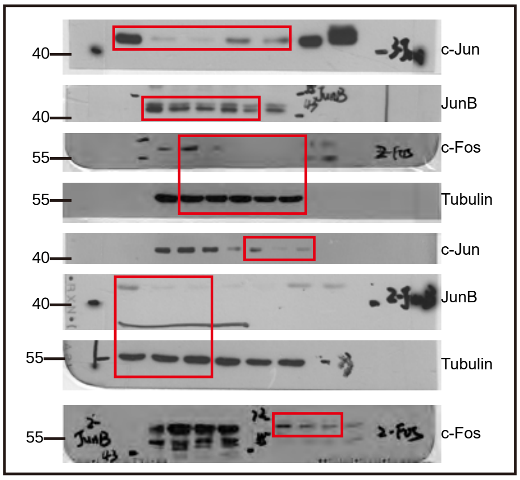

Supplementary Fig. 2b

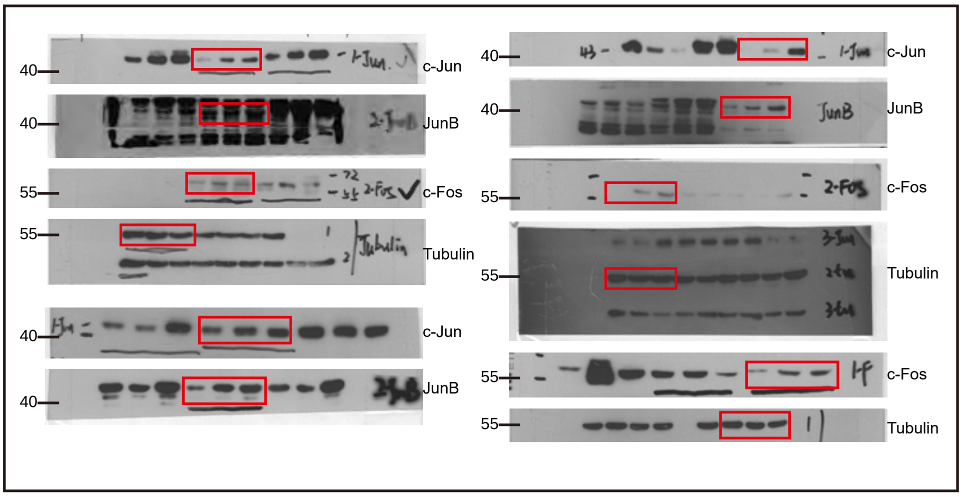

Supplementary Fig. 2c

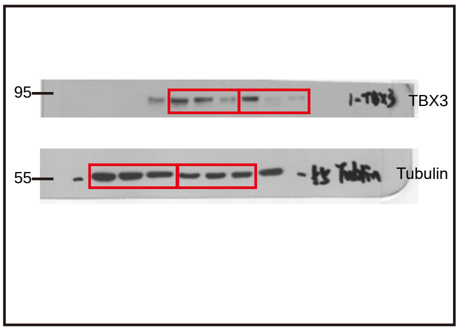

Supplementary Fig. 2d

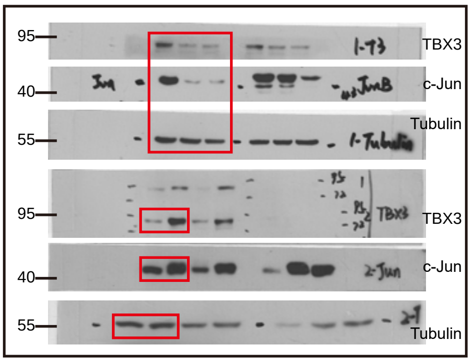

Supplementary Fig. 2e

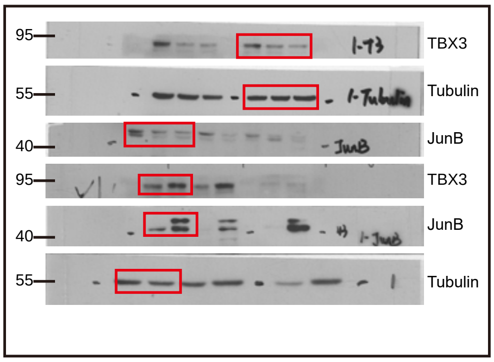

Supplementary Fig. 2f

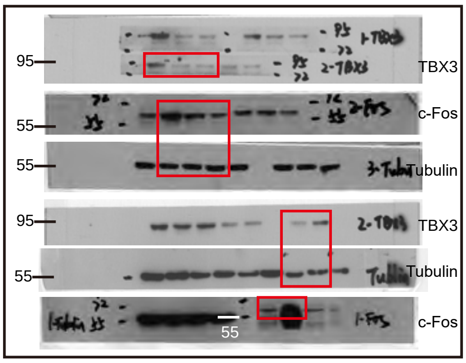

Supplementary Fig. 2g

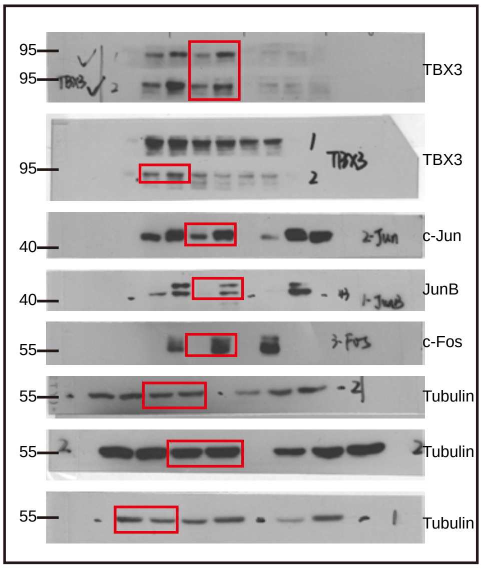

Supplementary Fig. 2i

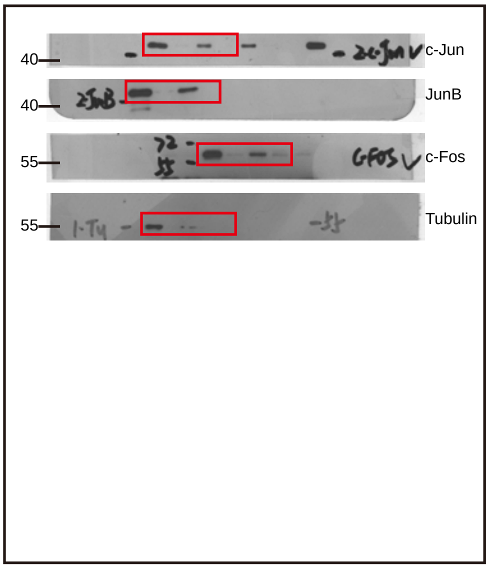

Supplementary Fig. 2j

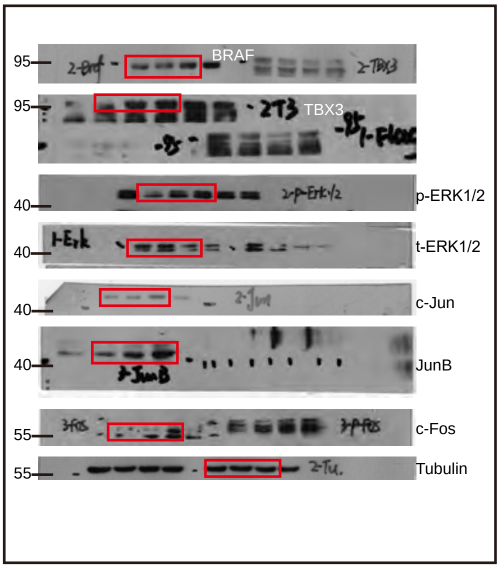

Supplementary Fig. 3h

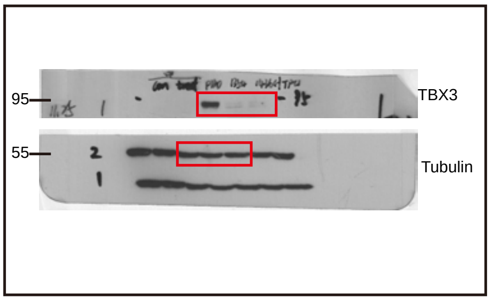

Supplementary Fig. 3i

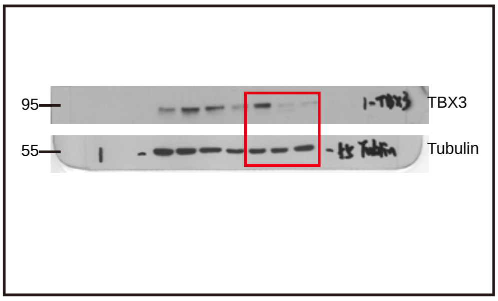

Supplementary Fig. 4b

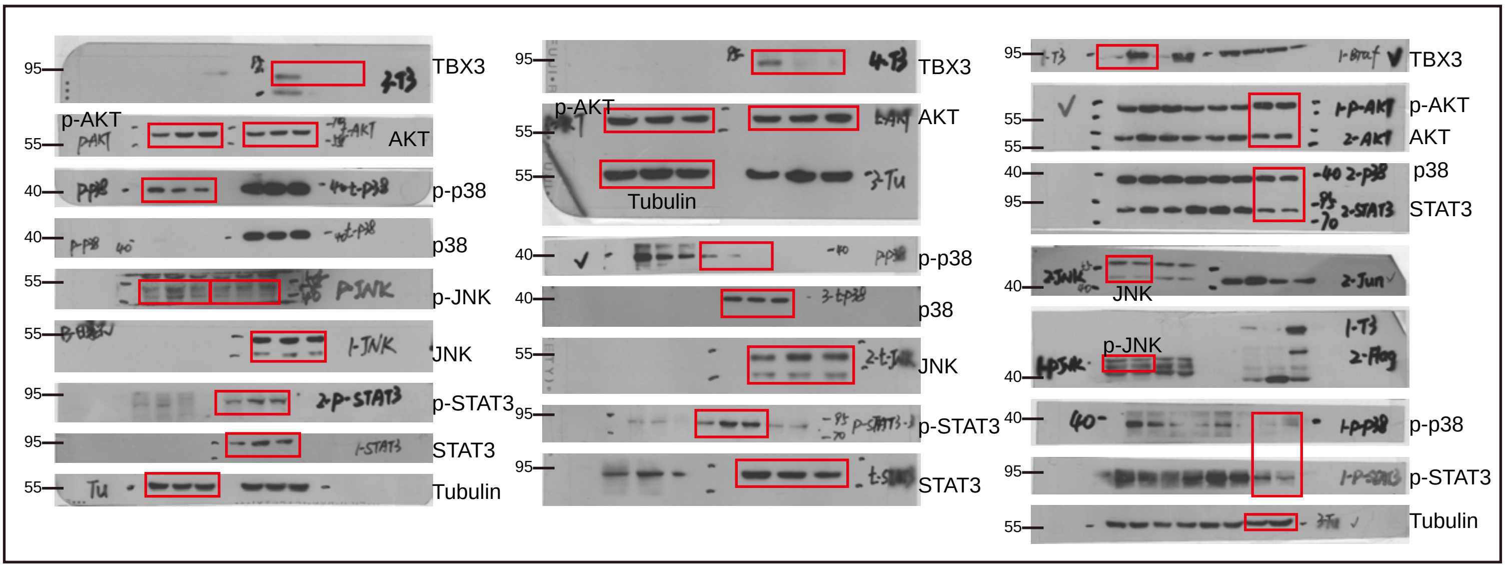

Supplementary Fig. 4f

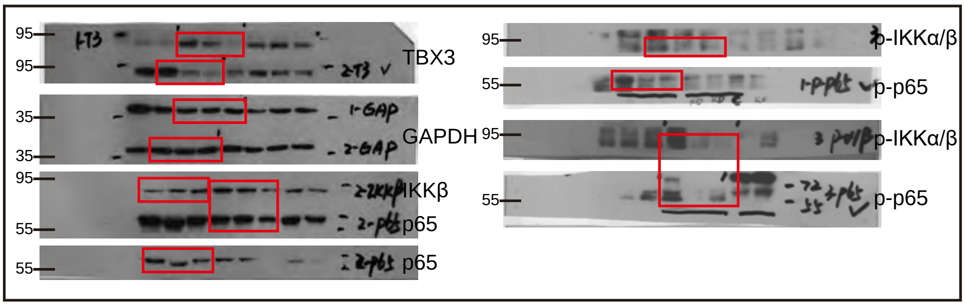

Supplementary Fig. 9a

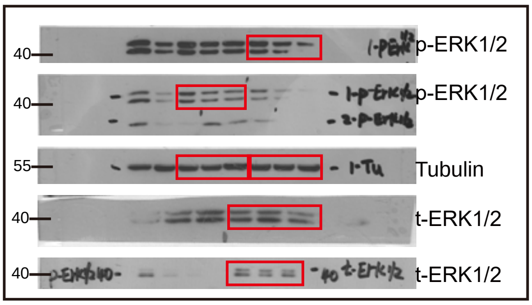

Supplement: Supplementary file 8 — Source Data [file 41467_2022_29000_MOESM8_ESM.zip › Uncropped immunoblots.pdf]
